# The dental phenotype of hairless dogs with *FOXI3* haploinsufficiency

**DOI:** 10.1038/s41598-017-05764-5

**Published:** 2017-07-14

**Authors:** Kornelius Kupczik, Alexander Cagan, Silke Brauer, Martin S. Fischer

**Affiliations:** 1 Max Planck Weizmann Center for Integrative Archaeology and Anthropology, Max Planck Institute for Evolutionary Anthropology, Deutscher Platz 6, 04103 Leipzig, Germany; 2 Institut für Spezielle Zoologie und Evolutionsbiologie mit Phyletischem Museum, Friedrich-Schiller-Universität, Erbertstrasse 1, 07743 Jena, Germany; 3 Department of Evolutionary Genetics, Max Planck Institute for Evolutionary Anthropology, Deutscher Platz 6, 04103 Leipzig, Germany; 4 Wellcome Trust Sanger Institute, Hinxton, Cambridge CB10 1SA UK

## Abstract

Hairless dog breeds show a form of ectodermal dysplasia characterised by a lack of hair and abnormal tooth morphology. This has been attributed to a semi-dominant 7-base-pair duplication in the first exon of the forkhead box I3 gene (*FOXI3*) shared by all three breeds. Here, we identified this *FOXI3* variant in a historical museum sample of pedigreed hairless dog skulls by using ancient DNA extraction and present the associated dental phenotype. Unlike in the coated wild type dogs, the hairless dogs were characterised in both the mandibular and maxillary dentition by a loss of the permanent canines, premolars and to some extent incisors. In addition, the deciduous fourth premolars and permanent first and second molars consistently lacked the distal and lingual cusps; this resulted in only a single enlarged cusp in the basin-like heel (talonid in lower molars, talon in upper molars). This molar phenotype is also found among several living and fossil carnivorans and the extinct order Creodonta in which it is associated with hypercarnivory. We therefore suggest that *FOXI3* may generally be involved in dental (cusp) development within and across mammalian lineages including the hominids which are known to exhibit marked variability in the presence of lingual cusps.

## Introduction

Intraspecific and interspecific variations in mammalian teeth are the result of changes to the genes, microRNAs and signalling pathways involved in dental development. In mice, for example, besides the inhibition of microRNAs different signalling levels of the ectodysplasin A (*EDA*) or Activin A proteins (*ACVR1*) result in different cusp numbers while sonic hedgehog (*SHH*) and fibroblast growth factor (Fgf3) also contribute to overall crown shape complexity^1–3^. These signalling pathways (EDA, Activin A, SHH and BMP4) involved in cusp formation, are linked by the FOXI3 transcription factor expressed in the dental lamina^4^. The *FOXI3* gene is a member of the forkhead box transcription factor gene family, which has important roles in hair, ear, jaw and dental development^5–7^, with its deletion leading to alterations in cusp pattering in the mouse^7, 8^. In the ferret (*Mustela putorius furo*) it has been shown that FOXI3 expression is involved in tooth initiation and replacement, which is suggested to be regulated by Activin rather than EDA^9^. A 7-bp duplication in the first exon of *FOXI3*, which causes a frameshift that results in a premature stop-codon, causes the semi-dominantly inherited phenotype of hairless dog breeds (known as Canine Ectodermal Dysplasia (CED))^5^. The Chinese Crested, Peruvian and Mexican hairless dog breeds are characterized by sparse or absent coat as well as missing or misshapen permanent teeth^5, 10^. Here we present the precise cusp patterning of the mandibular and maxillary deciduous premolar and permanent molars associated with the *FOXI3* heterozygous variant in a historical pedigreed skeletal collection of hairless and coated dogs. This unique sample derives from a breeding experiment of Ludwig Plate, the successor of Ernst Haeckel in Jena, from the beginning of the 20^th^ century, originally devised to study the heredity of hair and skin characteristics^11, 12^ (Figure S1).

## Results

### Dental phenotype

**Figure 1:**
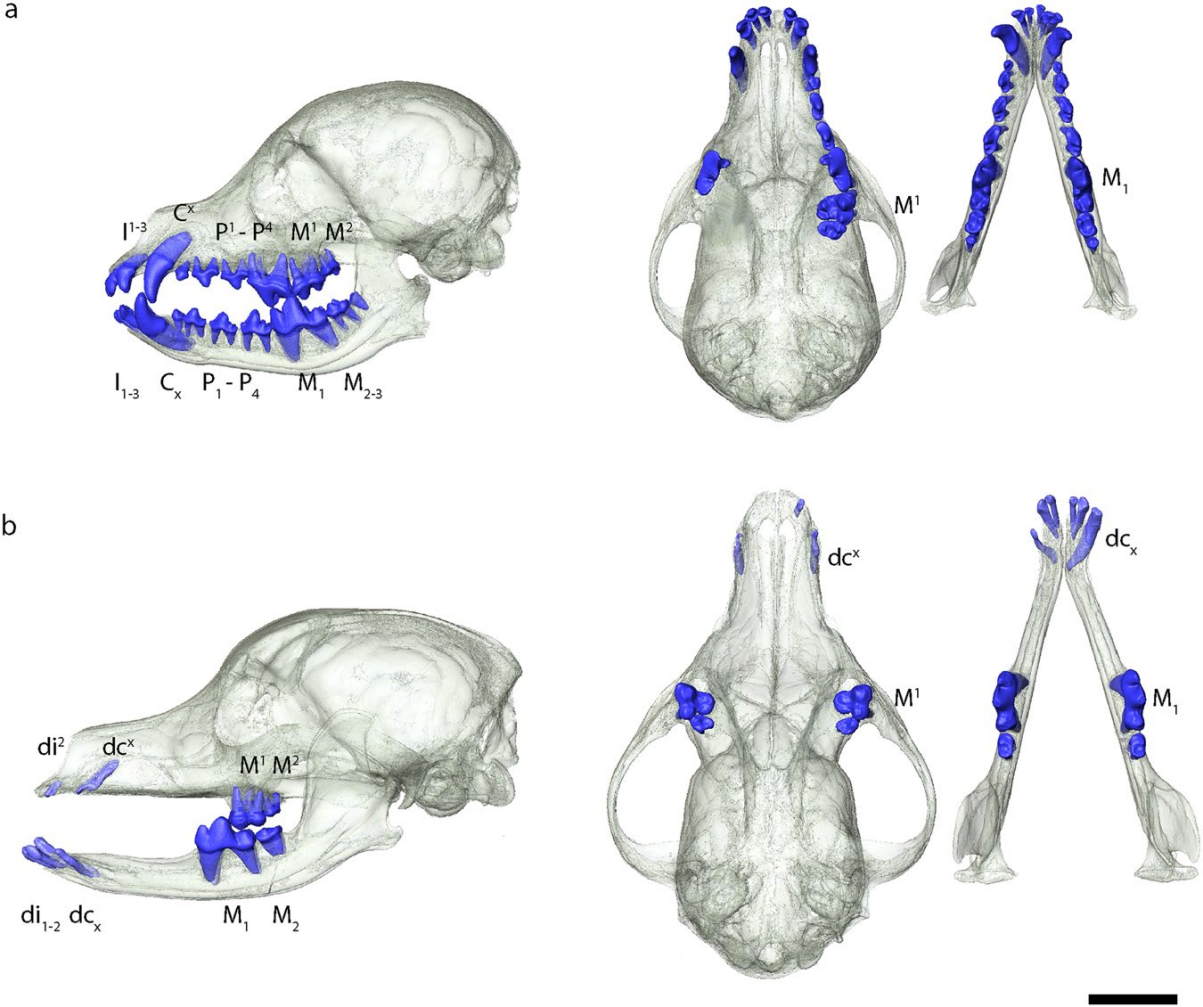
Dental phenotype in (**a**) normally coated dog (MAM1491) and (**b**) hairless dog (MAM1494). 3D renderings of skulls (transparent) and teeth in lateral (left) and superior view (right). Note missing premolars and canines in hairless type; only the molars and the deciduous incisors and canines are present. In the coated dog there are some maxillary postcanine teeth on the right side missing due to post mortem tooth loss. The black scale bar equals 5 cm.

To assess the dental phenotype (presence/absence of deciduous and permanent teeth as well as molar cusp pattern) of the 14 dogs (9 adults and 5 juveniles; 7 coated, 6 hairless and one patchy hair coat individuals; Table 1) we employed high-resolution micro-CT scanning of the dentitions and skulls as well as microscopic analysis of maxillary and mandibular molars. Among the adult individuals, the coated dogs had complete upper and lower dentitions with permanent teeth including first premolars (except MAM1492) and molars (Table 2; Fig. 1, Supplementary Fig. S2–S5). The lower third molars were missing in three out of four individuals. Specimen MAM1492 had a retained maxillary deciduous canine placed distal to the permanent canine (Supplementary Fig. S3). The three juvenile coated individuals showed a mixed dentition of erupted deciduous and unerupted permanent teeth still in development (incisors, canines and premolars as well as first premolar and first molar; Table 2; Supplementary Fig. S6–S8). In contrast, the adult and the juvenile hairless dogs entirely lacked the permanent mandibular and maxillary canines and premolars and to some extent the incisors, while fully developed deciduous canines and premolars were retained in the majority of these hairless dogs (Table 2; Fig. 1). In two adult hairless dogs (MAM1488 and 1490) the permanent mandibular and maxillary incisors were completely developed, while in the juvenile MAM2419 there were developing mandibular first and second incisor enamel caps (Supplementary Figs S9, S10 and S14). In three hairless individuals (MAM1494 and 1497) the right mandibular canines had a split crown, which could be the result of fusion with the third incisors and which was not present in these individuals (Supplementary Figs S11, S13). The phenotype of the patchy hair coat dog (MAM1489) was similar to that of the hairless dogs in terms of the congenital lack of both mandibular and maxillary permanent premolars while retaining the deciduous premolars (Table 2; Supplementary Fig. S15). In addition, the maxillary canines were rather small and resembled a deciduous canine while the mandibular canine was identified as a permanent one. There was also an agenesis of the M2s (mandibular and maxillary) and the mandibular M3s. Akin to two of the fully hairless specimens, the right mandibular canine crown was split although the third incisor was clearly visible. In all hairless and the patchy hair coat individuals the lower and upper first molars and largely the first premolars were present, while the third molars were missing in three out of five adult individuals (Table 2).

**Table 1:** Sample list.

Accession no	Sex	Age^1^	Coat	FOXI3 variant^2^	Specimen type
Mam1493	M	juvenile	Yes	—	Skull
Mam1495	M	juvenile	Yes	—	Skull
Mam1500	M	juvenile	Yes	+/+	Skull
Mam1491	M	adult	Yes	+/+	Skull
Mam1492	M	adult	Yes	+/+	Skull
Mam1499	F	adult	Yes	+/+	Skull
Mam1501	F	adult	Yes	—	Mandible only
Mam2419	F	juvenile	No	—	Wet specimen
Mam1488	F	adult	No	+/−	Skull
Mam1490	M	adult	No	+/−	Skull
Mam1494	M	adult	No	+/−	Skull
Mam1496	F	adult	No	+/−	Skull
Mam1497	F	adult	No	+/−	Skull
Mam1489	M	juvenile	patchy	+/−	Skull

^1^Adult: >4 months; permanent molars erupted; juvenile: <4 months; deciduous teeth erupted, some or all permanent molars unerupted. ^2^+/+ = Two copies of 119-bp allele present; +/− =  one copy of 119-bp allele and one copy of 126-bp allele present; − = no/insufficient DNA yield.

**Table 2:** Dental status (maxillary and mandibular) in coated and hairless dog sample.

Coat	Accession number		I1	I2	I3	C	P1	P2	P3	P4	M1	M2	M3
Coated	1493*	max.	(1)	(1)	(1)	(1)	0	(1)	(1)	(1)	1	0	
		mand.	(1)	(1)	(0)	(1)	1	(0)	(1)	(0)	1	0	0
	1495*	max.	(1)	(1)	(1)	(1)	1	(1)	(1)	(1)	1	0	
		mand.	(1)	(1)	(1)	(1)	1	(1)	(0)	(0)	1	0	0
	1500*	max.	(1)	(1)	(1)	(1)	1	(1)	(1)	(1)	1	1	
		mand.	(1)	(1)	(1)	(1)	1	(1)	(0)	(0)	1	1	0
	1491	max.	1	1	1	1	1	1	1	1	1	1	
		mand.	1	1	1	1	1	1	1	1	1	1	1
	1492	max.	1	1	1	1	1	1	1	1	1	1	
		mand.	1	1	1	1	0	1	1	1	1	1	0
	1499	max.	1	1	1	1	1	1	0	1	1	1	
		mand.	1	1	1	1	1	1	1	1	1	1	1
Hairless	1501	max.	Skull missing										
		mand.	1	1	1	1	1	1	1	1	1	1	1
	2419*	max.	0	0	0	0	1	(0)	(0)	(0)	0	0	
		mand.	(1)	(1)	0	0	0	(0)	(0)	(0)	1	0	0
	1488	max.	1	1	1	(0)	1	(0)	(0)	0	1	1	
		mand.	1	1	1	(0)	0	(0)	(0)	(0)	1	1	1
	1490	max.	1	1	(0)	(0)	1	0	0	0	1	0	
		mand.	1	1	1	(0)	1	0	0	0	1	0	0
	1494	max.	0	0	0	(0)	0	0	0	0	1	1	
		mand.	(0)	(0)	0	(0)	0	0	0	0	1	1	0
	1496	max.	(0)	(0)	0	(0)	1	0	0	0	1	1	
		mand.	(0)	(0)	0	(0)	0	(0)	0	0	1	1	0
	1497	max.	(0)	(0)	1	(0)	1	(0)	0	0	1	0	
		mand.	(0)	(0)	0	(0)	1	(0)	0	0	1	1	1
Patchy	1489*	max.	1	(1)	(1)	0	1	(0)	(0)	0	1	0	
		mand.	1	1	1	1	0	(0)	(0)	(0)	1	0	0

Presence and absence of deciduous and permanent teeth indicated by the following codes: 1 = fully developed permanent tooth or molar; (1) deciduous tooth and developing permanent tooth or molar; 0 = missing deciduous or permanent tooth; (0) = retained deciduous tooth and missing permanent tooth; max. = maxillary; mand. = mandibular. *Juveniles with mixed deciduous and developing permanent dentition.

**Figure 2:**
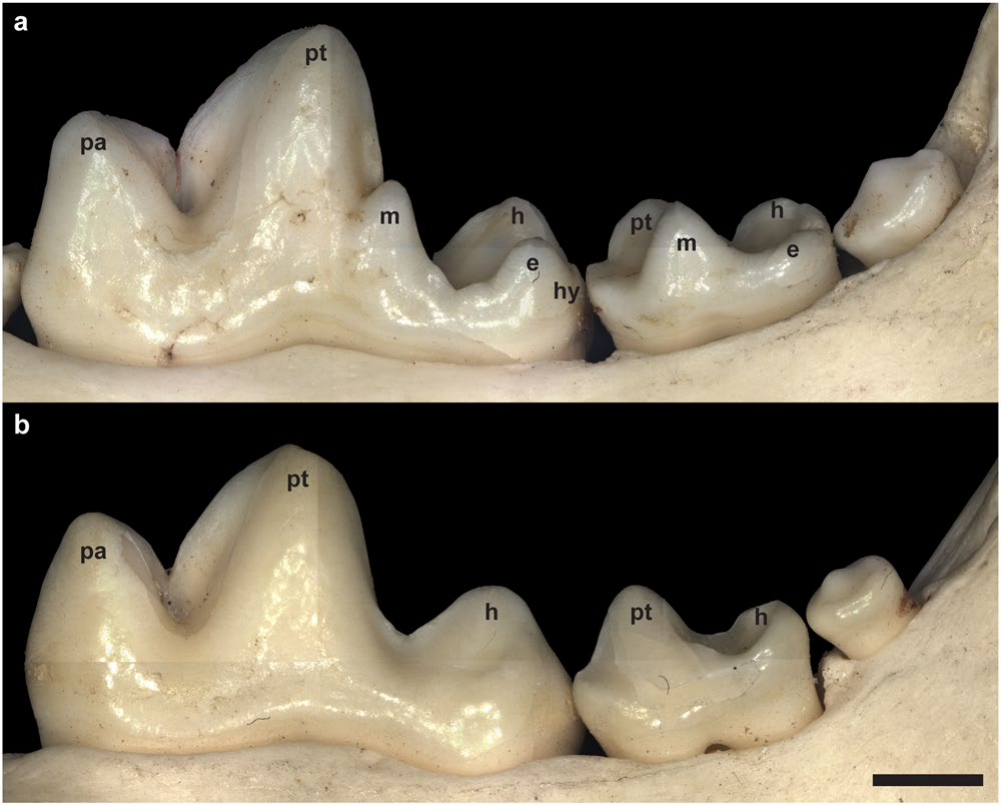
Mandibular right first, second and third molars in lingual view of (**a**) coated (MAM1491) and (**b**) hairless dog (MAM1497). Note the clearly developed metaconid (m), entoconid (e) and hypoconulid cusps (hy) in the coated dog which are lacking in the hairless dog. h = hypoconid, pa = paraconid, pt = protoconid. Teeth are shown in lingual view. Mesial is to the left. The black scale bar equals 3 mm.

**Figure 3:**
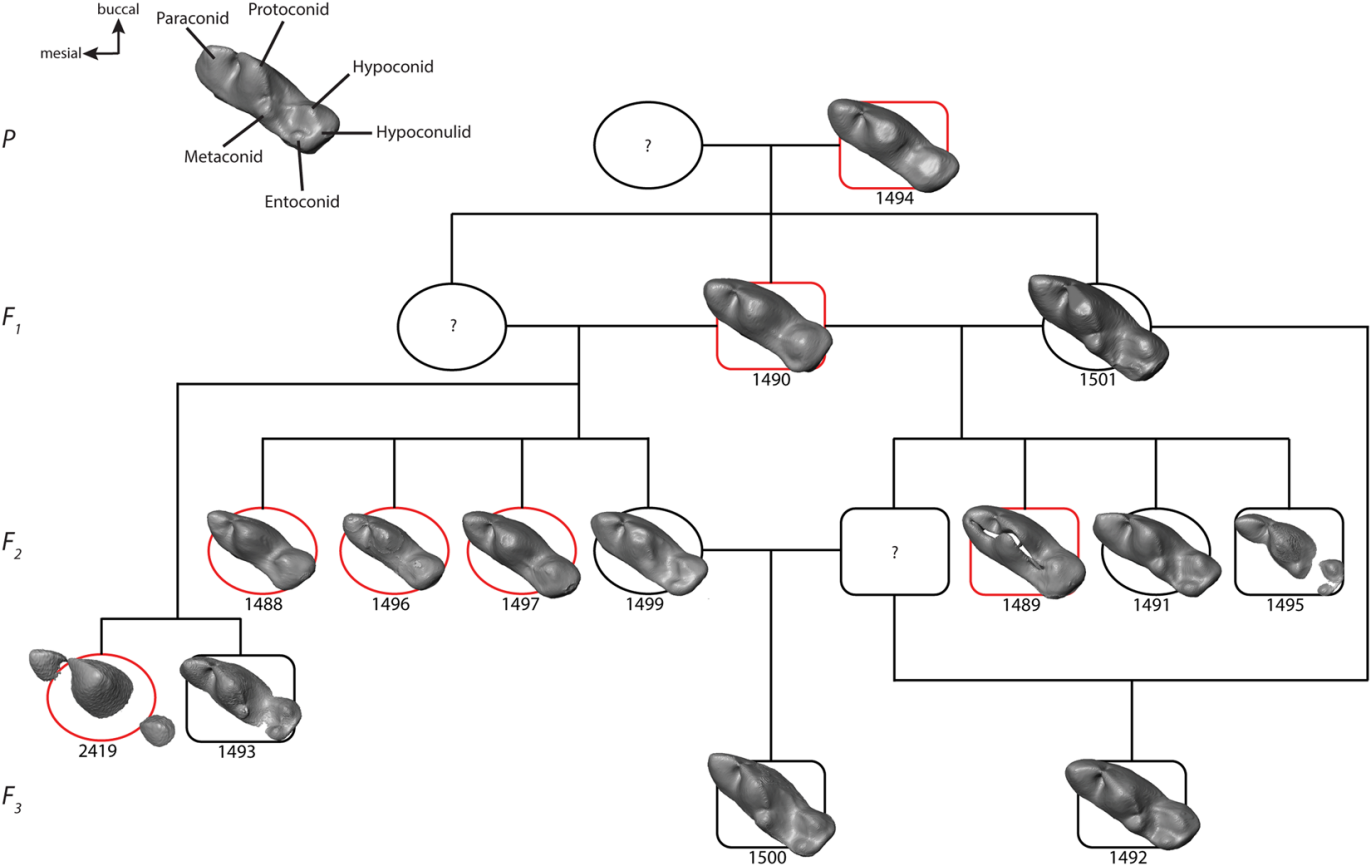
Lower first molar morphology mapped on genealogy of dog sample (genealogy from Plate, 1929). Circles are females and squares males. Red indicates hairlessness. ? = specimen missing from collection. A molar of a coated dog with normal cusp morphology is presented for comparison (top right).

**Figure 4:**
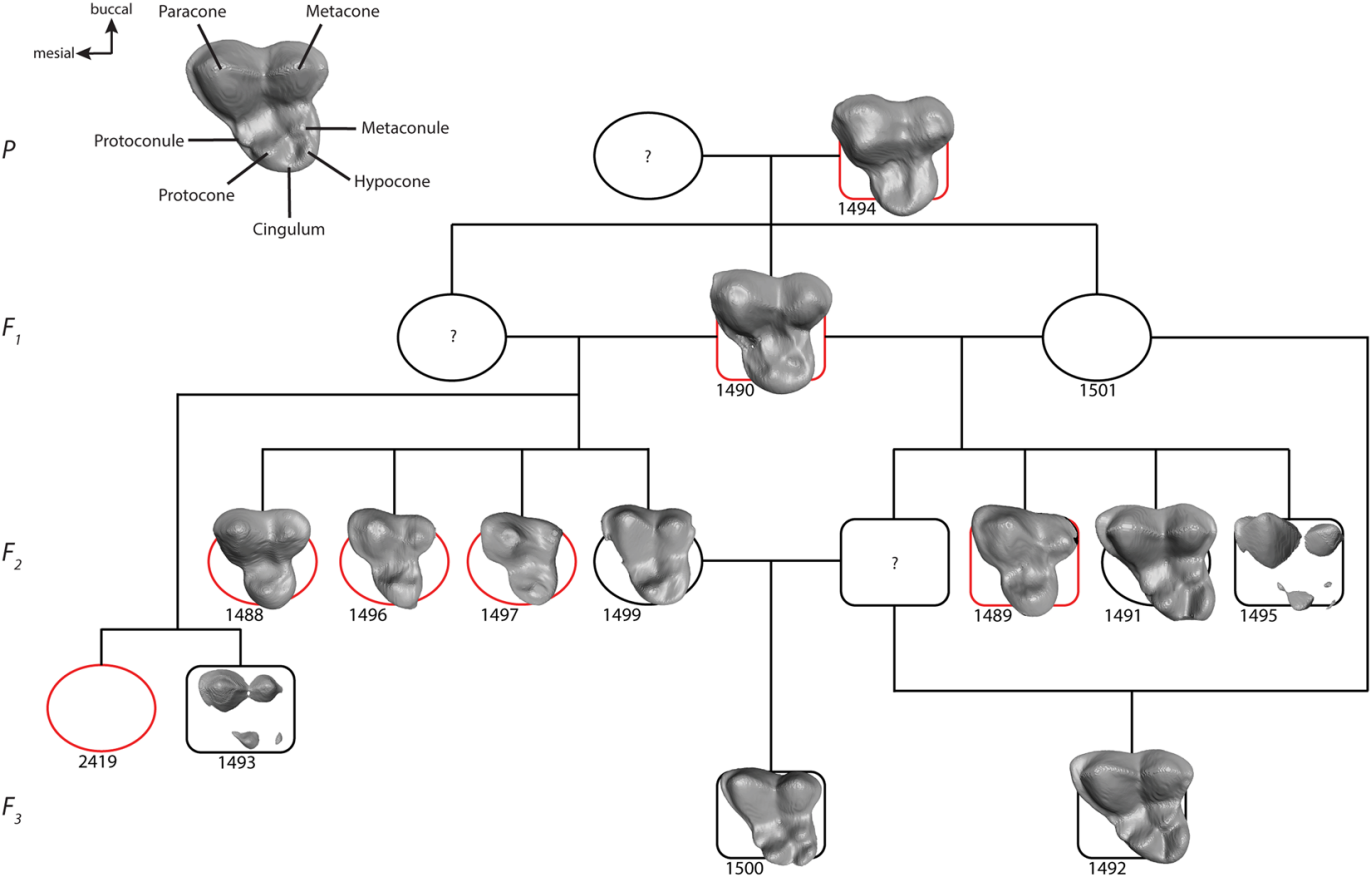
Upper first molar morphology mapped on genealogy of dog sample (see Fig. 2). Same symbols as in Fig. 2. The undeveloped molars of MAM 1501 and 2419 are not shown. A molar of a coated dog with normal cusp morphology is presented for comparison (top right).

**Figure 5:**
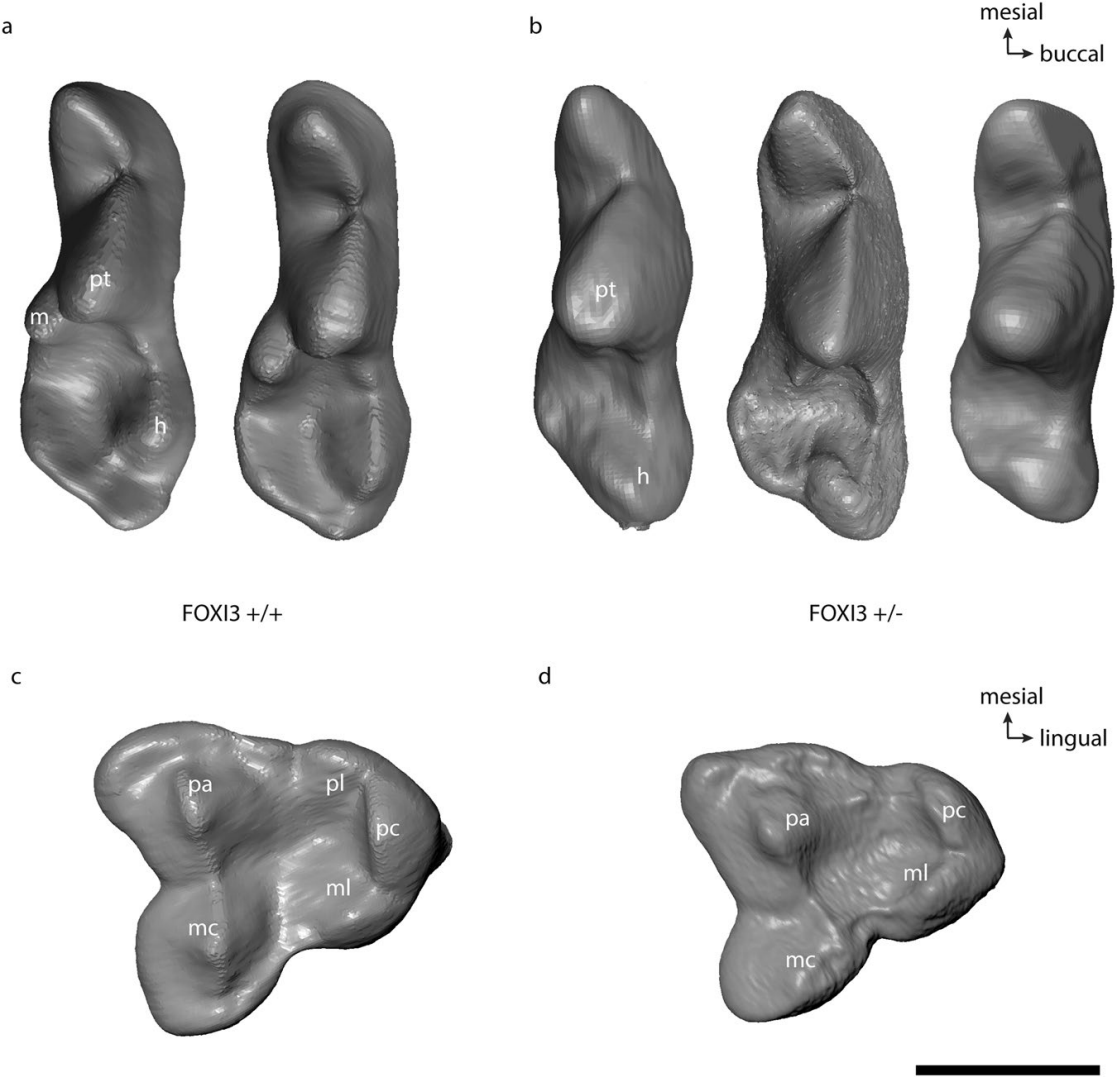
Occlusal morphology in mandibular (**a**,**b**) and maxillary (**c**,**d**) deciduous fourth premolars. (**a**) *FOXI3*+/+ variants (MAM1493, MAM1495) and (**b**) *FOXI3* +/− variants (MAM1488, MAM2419, MAM1489). Note that the metaconid is lacking and the hypoconid is distally displaced. (**c**) *FOXI3*+/+ variant (MAM1495), (**d**) *FOXI3* +/− variant (MAM2419). Note the size reduced metacone and the missing protoconule. All teeth are right side, except MAM1493 which is left and reflected here. Cusp abbreviation: h = hypoconid, m = metaconid, ml = metaconule, pa = paracone, pc = protocone, pl = protoconule, pt = protoconid,. Scale bar equals 5 mm.

In terms of molar cusp morphology, the teeth in the hairless and patchy hair coat dogs were characterised by a reduced cusp number compared to dogs with a full coat. Thus, in these seven individuals the mandibular first molars lacked the metaconid, entoconid and hypoconulid cusps, leaving only the hypoconid in the talonid part of the tooth (Fig. 2). This phenotype was present both within a litter and across the generations (Fig. 3). Likewise, in the maxillary first molar of hairless dogs the metacone was reduced in size and the protoconule and metaconule cusps were not developed and a cingulum was missing (Fig. 4). In addition, in the hairless dogs both mandibular and maxillary M2s only the protoconid/hypoconid and paracone cusps, respectively, were developed and the roots were fused and/or reduced in number (Fig. 2, Supplementary Fig. S16). Akin to the altered morphology of the permanent molars in hairless dogs, the deciduous mandibular fourth premolars of three individuals (MAM1488, MAM2419, MAM1489) have a reduced metaconid cusp, and the hypoconid cusp was distally displaced (Fig. 5a,b). Moreover, in the maxillary deciduous fourth premolar the metacone is reduced and the protoconule is not developed in MAM2419 (Fig. 5c,d).

The mandibular first molars of the hairless dogs (n = 6; x = 15.08 mm, min = 13.1 mm, max = 16.71 mm) were mesiodistally shorter than those in the coated dogs (n = 7; x = 16.06 mm, min = 14.52 mm, max = 18.11 mm), although this difference was statistically not significant (Mann-Whitney U-Test U = 14, z = −0.929, p = 0.353) (Supplementary Table S1). In addition, the M1 talonid was somewhat (though not significantly) greater in the hairless dog phenotype than in the coated dog phenotype indicating that the hypoconid was enlarged in hairless dogs (Supplementary Table S2).

### Genotype

We successfully extracted and amplified DNA from the bony ear region of 10 out of the 14 specimens (Table 1; specimen MAM1501 was not sampled at all). We were unable to amplify (though extract) DNA from the skulls of two coated puppies (MAM1494 and 1495), and it was not possible to extract DNA from a soft tissue sample of an alcohol-fixed hairless juvenile (MAM2419). DNA yields were generally low, as expected when extracting from bone of museum specimens, and ranged from 30–70 ng/microliter. Using previously published primers^5^ polymerase chain reaction (PCR) results showed that in six individuals with the CED phenotype (missing coat and teeth including loss of molar cusps; see above), including the one with patchy hair coat, were heterozygous for the 7-bp duplication in the first exon on *FOXI3*, corroborating an earlier study^5^. These results implicate the *FOXI3* alteration as underlying the developmental abnormalities shared among the hairless dogs in this pedigree.

## Discussion

Although the associated dental phenotype of hairless dog breeds (lack of teeth; conical crowns with a decreased number of cusps) has been alluded to before^5^, we show for the first time the association of the *FOXI3* variant with a specific cusp pattern in teeth of the primary dentition (deciduous premolars and permanent molars) in a species other than the mouse model. Specifically, the haploinsufficiency of *FOXI3* leads to an incomplete development of the lingually positioned cusps in the trigon(id) and talon(id) parts of both upper and lower molars and deciduous fourth premolars, respectively. As a result, the mandibular first molars in the hairless dogs are mesiodistally shorter than those in the coated dogs. Our observation on the *FOXI3* +/− molar phenotype is confirmed by the results of a study on *Foxi3* conditional knockout mice showing that *Foxi3* is expressed on the lingual side of the developing lower molars between the bud and cap stages^7^. While in mice it appears that a single copy of *Foxi3* is sufficient for proper cusp formation, our results show that having only a single functional copy of *Foxi3* results in haploinsufficiency in dogs. The findings of our study are in accordance with the patterning cascade model of cusp development which predicts that the size, shape and location of the first-forming cusps are less variable than in later-forming cusps^13, 14^. Thus, in the first mandibular molar, the development of the tallest cusp (protoconid) begins first, and continued downward growth and folding in the epithelium form the smaller cusps (paraconid, metaconid, hypoconid, entoconid and hypoconulid). Our observation on the cusp morphology of the distal part of primary postcanine teeth in hairless dogs therefore suggests that *FOXI3* is involved in the later forming secondary (and distal) enamel knots, i.e. the precursors of the cusps.

In mice, *Foxi3* is a known target of Eda, Activin A, Shh (upregulators) and Bmp4 (downregulator), and it suppresses epithelial cell differentiation in the developing molar^7, 8^. Our findings may specifically help to clarify the function of the Eda pathway in dental development. While a lack of expression of Eda (as in the natural mutant Tabby mouse or dogs with X-linked ectodermal dysplasia) leads to a talonid with a shallow shelf lacking well-defined cusps, low levels of Eda result in a single cusp on the talonid and normal Eda levels result in the formation of the hypoconid and the entoconid cusps^1, 15, 16^. Concomitantly, Foxi3, as a downstream target of Eda, is downregulated in *Eda*-null mice but overexpressed in *Eda*-overexpressing (K14-*Eda*) mice^8^. The EDA/FOXI3 pathway therefore appears to affect crown formation (cusp number and crown size) in a dose-dependent manner. Although the dentition of mice is specialised in terms of the number of teeth as well as the mode of tooth replacement compared to dogs, we hypothesise that this dental developmental pathway is conserved across mammals, and we speculate that evolutionary character states are the result of different thresholds of these signalling molecules^1^.

Interestingly, the observed phenotype of the *FOXI3* +/− variants with one central and two smaller cusps mesially and distally mimics some of the molar phenotypes found among extant and extinct Canidae (e.g. ^†^Borophaginae, ^†^Hesperocyoninae). Moreover, the tri-cusped morphology of the *FOXI3 +*/− dogs resembles that of the postcanine teeth of some seals (Phocidae). For example, Lake Ladoga seals (*Phoca hispida ladogensis*) have teeth that bear between three and five cusps, and this variation has been attributed to changes in the activator–inhibitor dynamics of the enamel knot development^13, 17^. Among the Canini (Canidae without foxes), hypercarnivorous taxa such as the dhole (*Cuon alpinus*), hunting dog (*Lycaon pictus*) and bush dog (*Speothos venaticus*) tend to have lower M1s with a single, enlarged hypoconid (known as a trenchant heel) concomitant with a reduced entoconid and metaconid compared to hypocarnivorous forms^18–20^. Likewise, the upper M1 of these hypercarnivorous taxa has a protocone only, while the hypocarnivorans also feature a hypocone. The trenchant talonid evolved several times independently in several families of the Carnivora and the extinct, polyphyletic Creodonta^19^, and we propose that this morphology is the result of a low level expression of the *FOXI3* gene during talonid development.

Among primates, species within the African great ape/human clade (including fossil hominins) also have a variable presence of the hypoconulid (or cusp 5) on the lower molars or even possess accessory molar cusps (as a result of additional secondary enamel knots) such as Carabelli’s cusp, a cusp 6 (C6) or cusp 7 (C7). While the Carabelli’s cusp occurs on the mesiolingual aspect (protocone) of maxillary molars, the C6 is located between the hypoconulid and the entoconid and the C7 between the metaconid and entoconid of the mandibular molars^21^. The expression of these lingually positioned accessory cusps has been associated with an enlarged overall crown size, the morphology of adjacent cusps and extended crown formation time^22–24^. Although our knowledge of the involvement of FOXI3 in dental development is limited to mice and ferrets, in the light of the findings of our study on dogs we suggest that variations in the *EDA*/*FOXI3* pathway may also be responsible for variations in hominid dental morphology. In fact, some support for this hypothesis comes from two reports on human patients with a sequence change in *FOXI3* published in the DECIPHER database (http://decipher.sanger.ac.uk/)^25^. One patient (no 252352) with a *FOXI3* deletion has among other abnormalities widely spaced and abnormally shaped teeth, while another patient (no. 328753) with *FOXI3* gene duplication is reported to lack some teeth (oligodontia). The above examples and the present study suggest the communality of *FOXI3* in dental development across several mammalian orders and highlight the need for further investigation.

## Materials and Methods

### Sample

The samples originated from a breeding experiment by German zoologist Ludwig Plate who studied the heredity of hair, skin and skeleton-dental characteristics in four generations of bred dogs between 1914 and 1919^11, 12^. The founding (parental) generation consisted of a male hairless dog (originally from Ceylon) and a female coated dachshund resulting in 33 animals over three filial generations of which one male F2-animal was backcrossed three times. Of the original 35 animals, the macerated skulls of 13 adult and juvenile individuals and one alcohol fixed whole body specimen of a two months old juvenile housed at the Phyletisches Museum of the Friedrich Schiller University Jena were available for study (Table 1). Of these, six individuals were described to completely lack a coat except for the top of the head, the feet and the tip of the tail^11^. One individual (accession number MAM1489) was also reported as hairless except for the head, neck and shoulder region as well as the anterior surfaces of the limbs^11^.

### CT scanning and microscopic analysis

The specimens were CT scanned with a BIR ACTIS 225⁄300 high-resolution industrial µCT scanner (Varian Medical Systems, Palo Alto, CA, USA) housed at the Department of Human Evolution, Max Planck Institute for Evolutionary Anthropology, Leipzig at 130 kV and 100 µA using a 0.5 brass filter. The isometric voxel size was 37 µm (juvenile MAM2419), 64 µm (all other puppies) and 91 µm (all adults). Segmentation of the teeth and the surrounding bone was performed in Avizo 7.1 (FEI, Hillsborough, OR, USA). Primary and replacement teeth were differentiated based on size and shape of the crowns and roots. In addition, high resolution photos using a Smartzoom 5 digital microscope (Zeiss, Jena, Germany) with a 1.6x lens were taken of the teeth of selected specimens.

### DNA extraction

To obtain DNA from these specimens we followed published extraction protocols^26, 27^. Briefly, bone powder was removed using a dentistry drill (Emax Evolution, Nakanashi, Tochigi, Japan) at the lowest speed setting from the bulla tympanica of the macerated skulls of 12 individuals. Approximately 2 g of bone was drilled into a fine powder per sample. Approximately 50 mg of bone powder combined with 1 mL of extraction buffer (0.45 M EDTA, 0.25 mg/mL Proteinase K, pH 8.0; AppliChem GmbH, Darmstadt, Germany and SIGMA- Aldrich, St. Louis, MO, USA, respectively) was used for each DNA extraction. Bone powder was resuspended by vortexing and rotated overnight at 37 °C. The remaining bone powder was subsequently pelleted by centrifugation (Centrifuge 5810 R, Eppendorf, Hamburg, Germany). The supernatant was added to a binding buffer (5 M Guanidine hydrochloride, 40% Isopropanol, 0.05% Tween 20; SIGMA- Aldrich, St. Louis, MO, USA) and placed in a custom extension reservoir-MinElute assembly (MinElute PCR Purification kit, Qiagen, Hilden, Germany). This binding apparatus was then centrifuged, followed by two wash steps. The MinElute column was then spun-dry and eluted using TET buffer (final concentrations: 1 mM EDTA, 10 mM Tris-HCL, 0.05% Tween 20; pH 8.0; AppliChem GmbH, Darmstadt, Germany) pippetted directly onto the silica membrane, followed by an incubation period and subsequent centrifugation. This elution step was repeated in order to collect the remaining DNA from the column. In the alcohol-fixed specimen (MAM2419) a 2 × 1 cm large skin section was cut out of the abdominal region for DNA sampling. We used the same extraction protocol as for the bone samples but did not detect any DNA and thus excluded the sample from further analysis.

### PCR

To test for the presence or absence of the 7-bp duplication in the first exon of *FOXI3* putatively underlying the hairless phenotype we performed PCR on the DNA extracts with primers designed to amplify this region. We used the same primers and PCR protocol as in ref. 5, including the use of Amplitaq Gold 360 Master Mix with a GC enchancer (Applied Biosystems, Foster City, CA, USA) due to the high GC-content of the target region. Coated dogs were expected to carry two copies of the functional 119-bp allele. If the alteration underlying the CED phenotype in these dogs is identical to other affected breeds we expected these individuals to be carry one copy of the 119-bp allele and one copy of a 126-bp allele resulting from a 7-bp insertion in the first exon of *FOXI3*. For positive controls we used commercially available wild type dog genomic DNA from one male and one female individual (Zyagen, San Diego, CA). Neither of the control samples is from a hairless breed. Fragment size was assessed using the 3730 DNA capillary sequencer (Applied Biosystems, Foster City, CA, USA) and results were analysed with the GeneMapper v3.7 software (Applied Biosystems, Foster City, CA, USA).

## Electronic supplementary material

Supplementary information
