# Supplementary material for: The dental phenotype of hairless dogs with FOXI3 haploinsufficiency
Source: Sci Rep. 2017 Jul 14;7:5459. doi: 10.1038/s41598-017-05764-5 (PMC5511229; doi:10.1038/s41598-017-05764-5)
Supplement: Supplementary file 1 — Supplementary information [file 41598_2017_5764_MOESM1_ESM.pdf]

Supplementary information

The dental phenotype of hairless dogs with FOXI3 haploinsufficiency

Kornelius Kupczik[1,2*], Alexander Cagan[3,4], Silke Brauer[1], Martin S. Fischer[2]

[1] Max Planck Weizmann Center for Integrative Archaeology and Anthropology, Max Planck Institute for Evolutionary Anthropology, Deutscher Platz 6, 04103 Leipzig, Germany.

[2] Institut für Spezielle Zoologie und Evolutionsbiologie mit Phyletischem Museum,

Friedrich-Schiller-Universität, Erbertstrasse 1, 07743 Jena, Germany.

[3] Department of Evolutionary Genetics, Max Planck Institute for Evolutionary Anthropology, Deutscher Platz 6, 04103 Leipzig, Germany.

[4] Wellcome Trust Sanger Institute, Hinxton, Cambridge CB10 1SA, UK.

[*]Corresponding author: kornelius_kupczik@eva.mpg.de

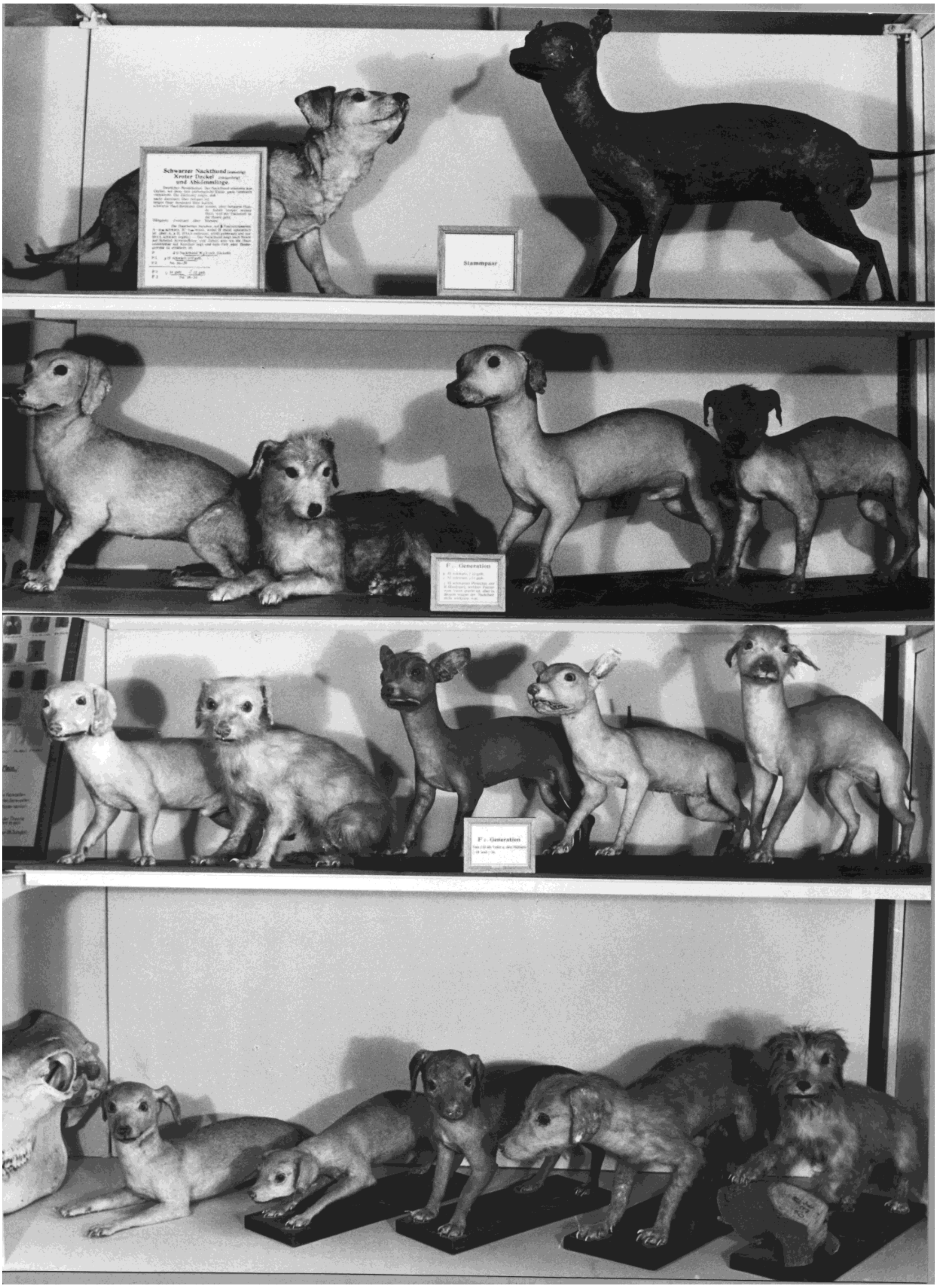

Supplementary Fig. S1: Historical photo of the museum’s show case with taxidermy specimens of the hairless dog experiment. The founding generation with a female coated dog (left) and a male hairless dog (right) is on the top. The F1 and F2 generations are second and third from top, respectively (Archive of Phyletisches Museum, Jena).

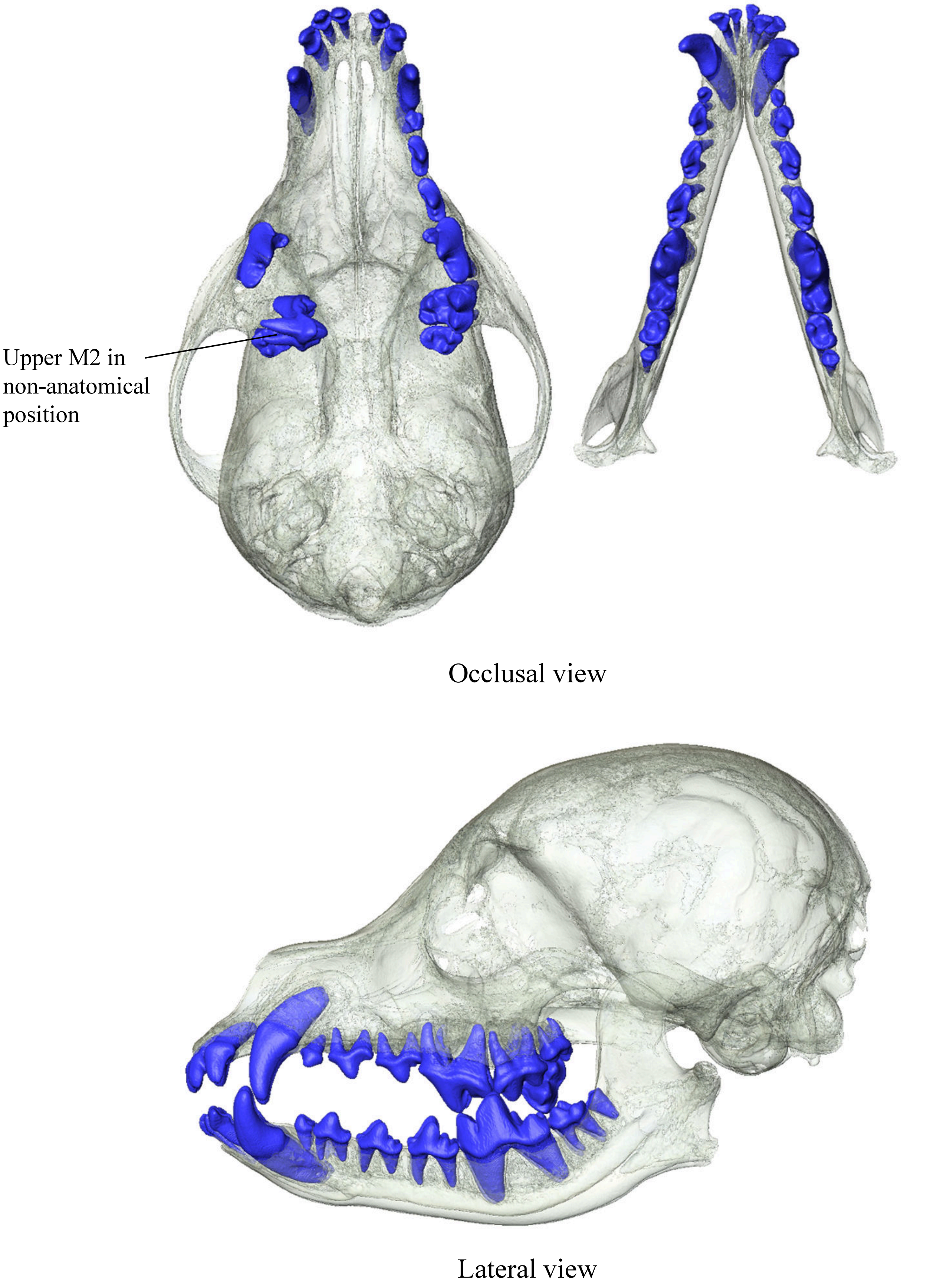

Supplementary Figure S2. Skull MAM1491, coated, complete permanent dentition

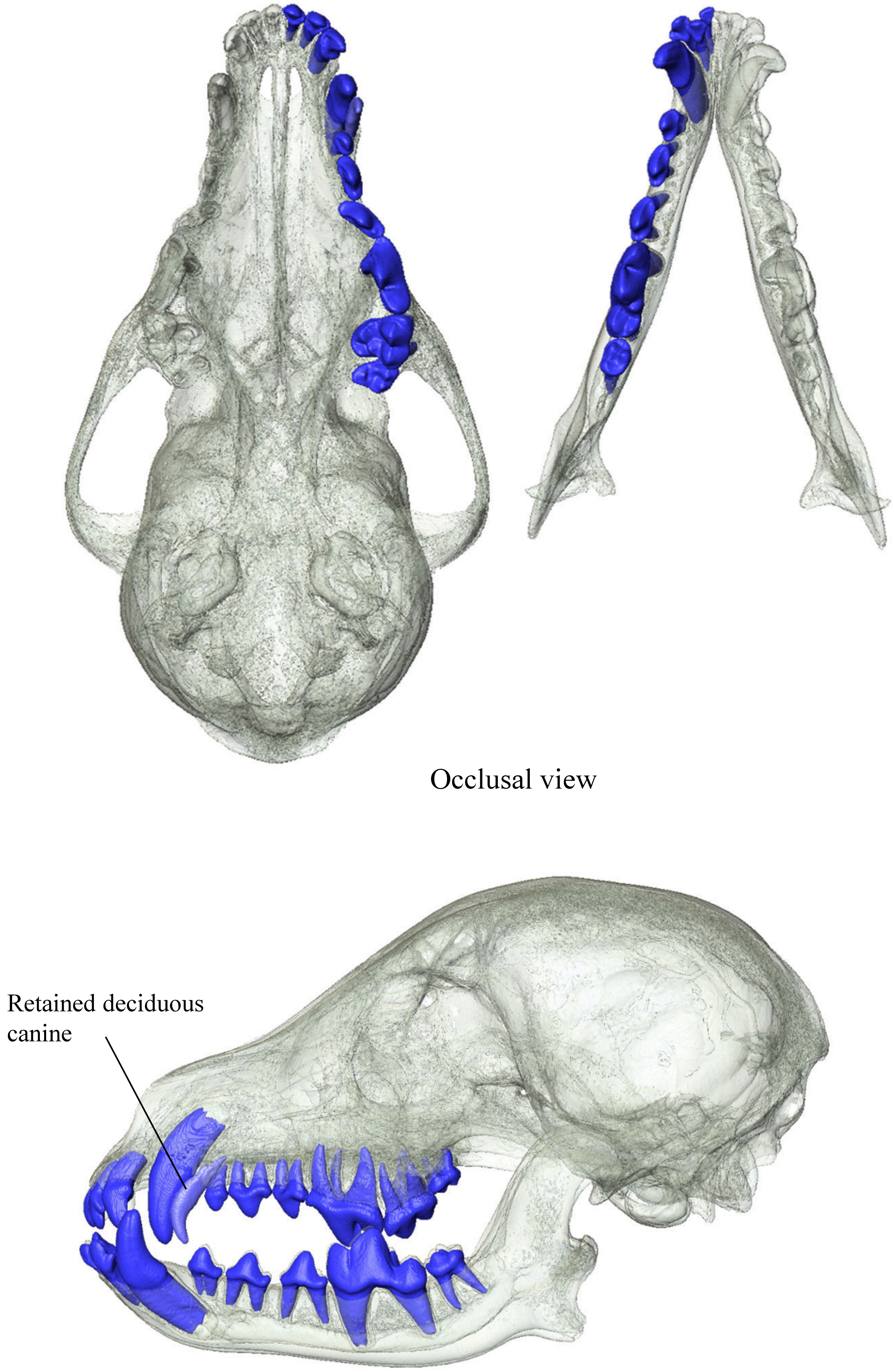

Supplementary Figure S3. Skull MAM1492, coated, complete permanent dentition

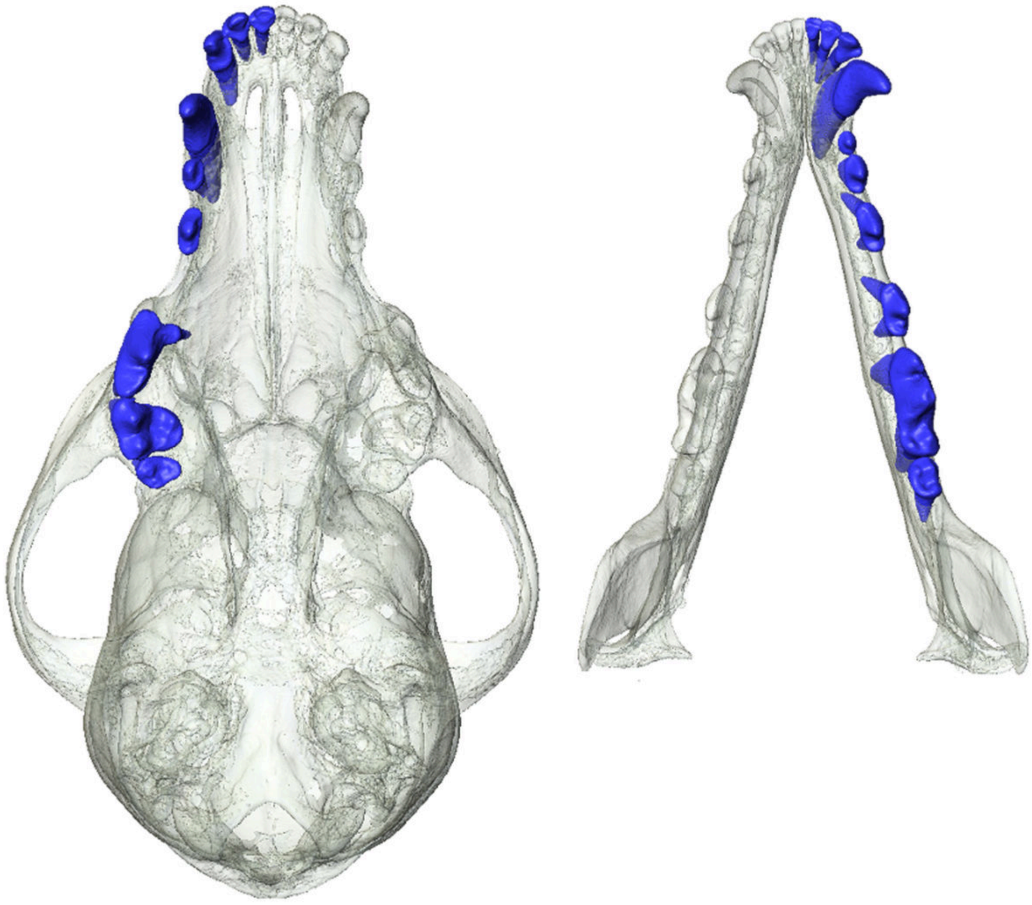

Occlusal view

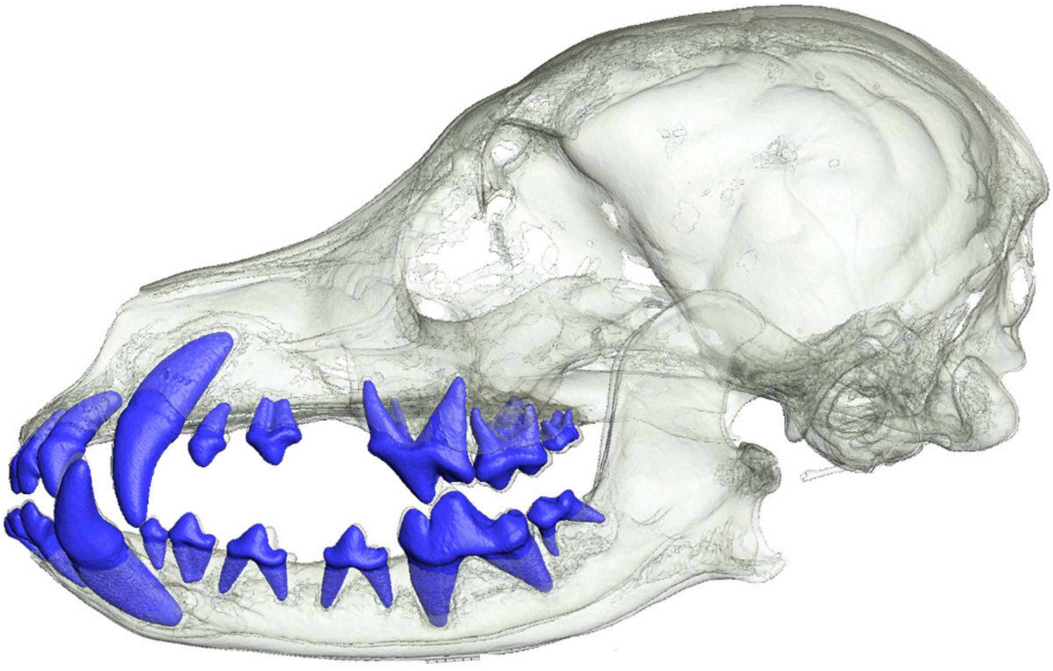

Lateral view

Supplementary Figure S4. Skull MAM1499, coated, complete permanent dentition

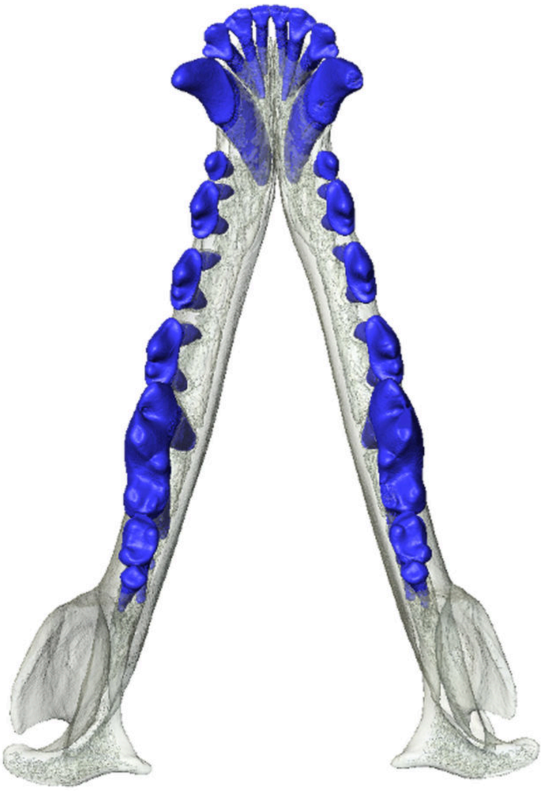

Occlusal view

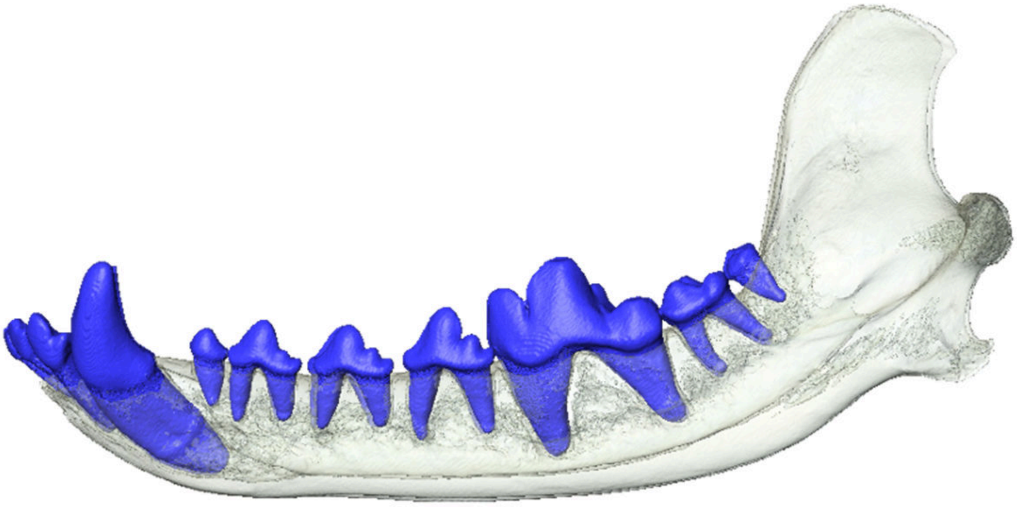

Lateral view

Supplementary Figure S5. Mandible MAM1501, coated, complete permanent dentition

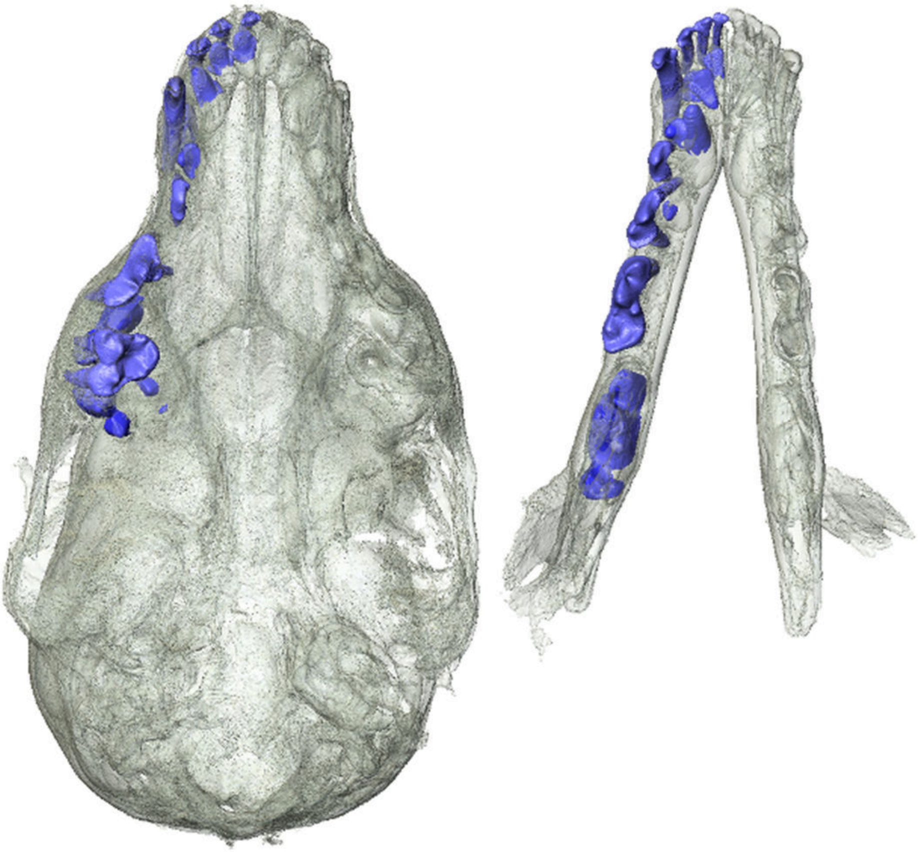

Occlusal view

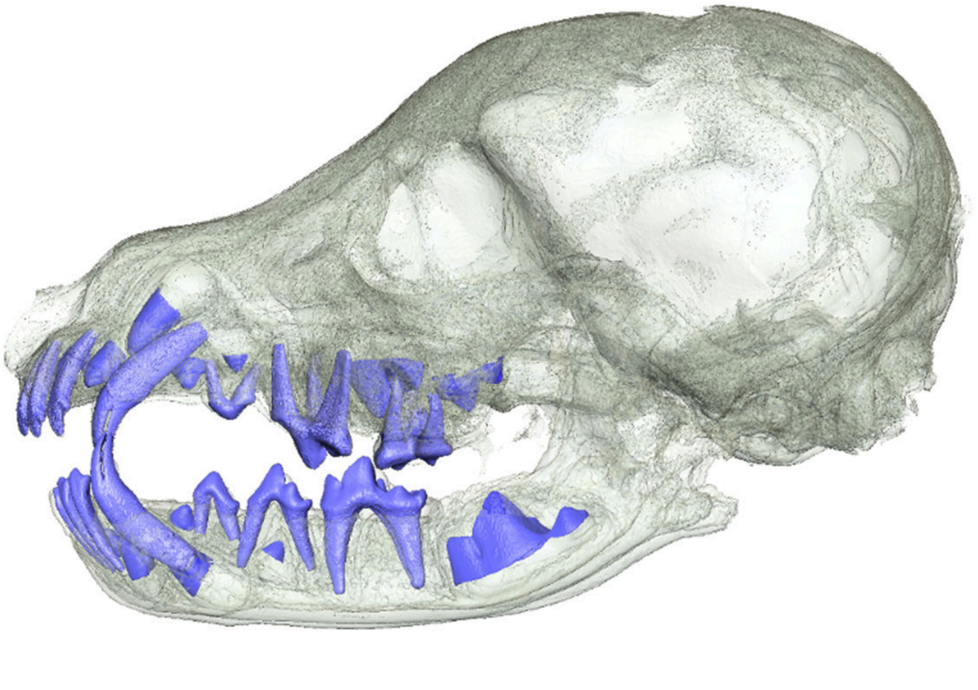

Lateral view

Supplementary Figure S6. Skull MAM1493, juvenile, coated, mixed dentition

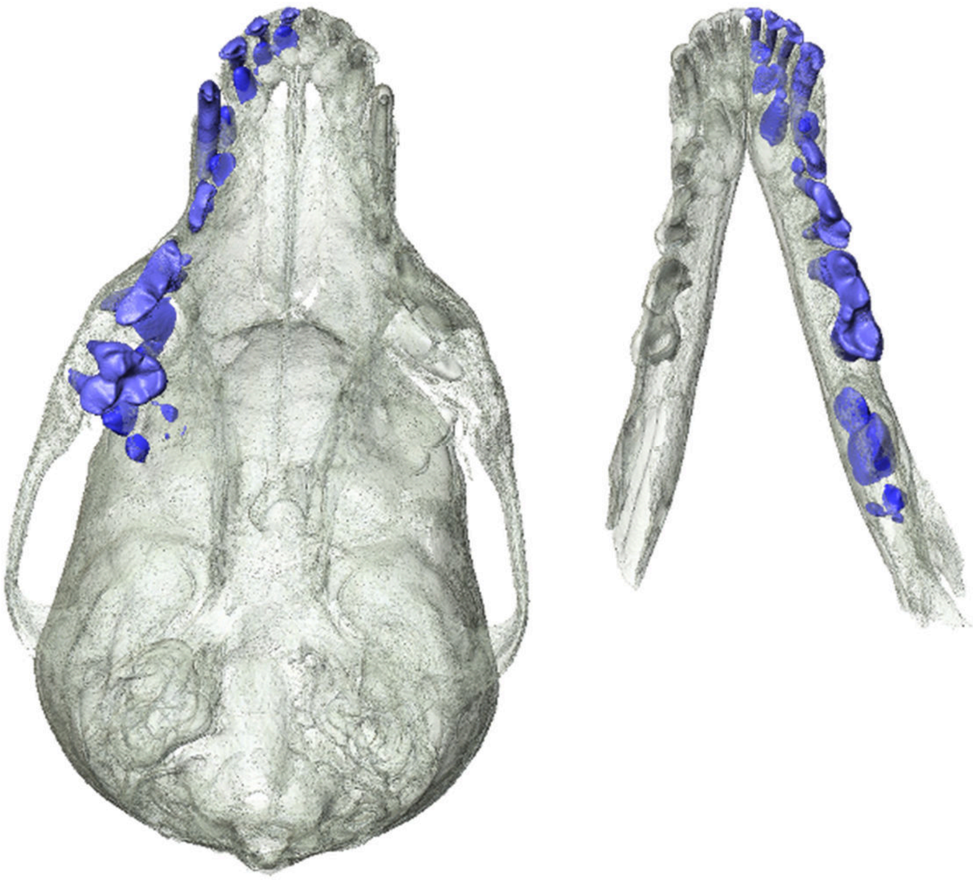

Occlusal view

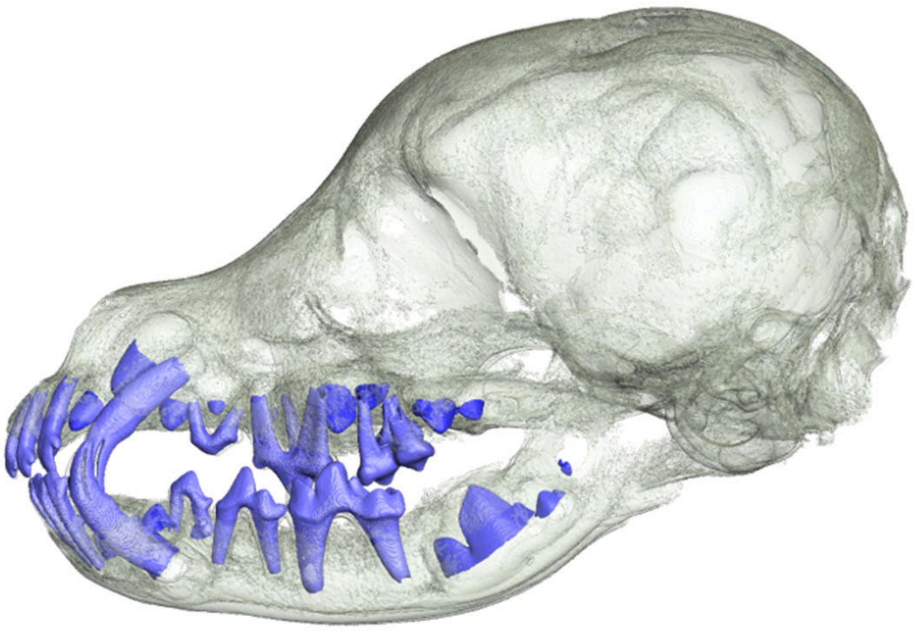

Lateral view

Supplementary Figure S7. Skull MAM1495, juvenile, coated, mixed dentition

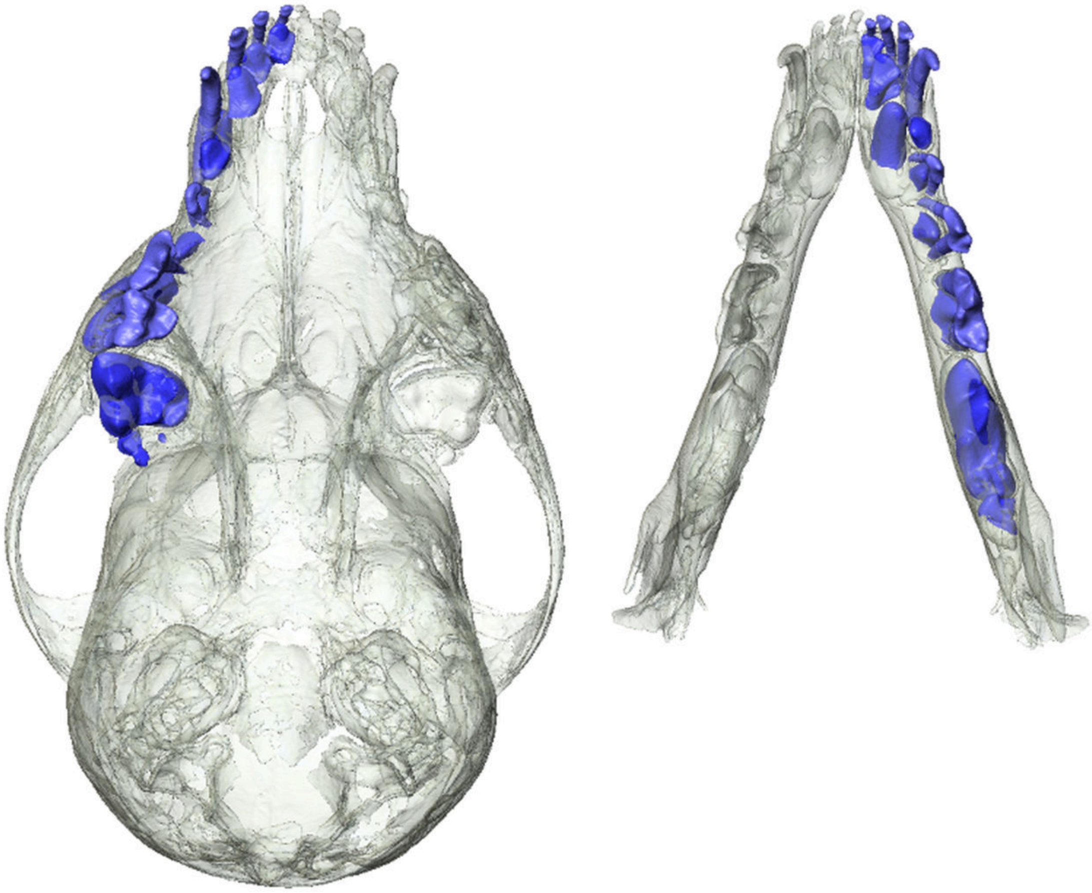

Occlusal view

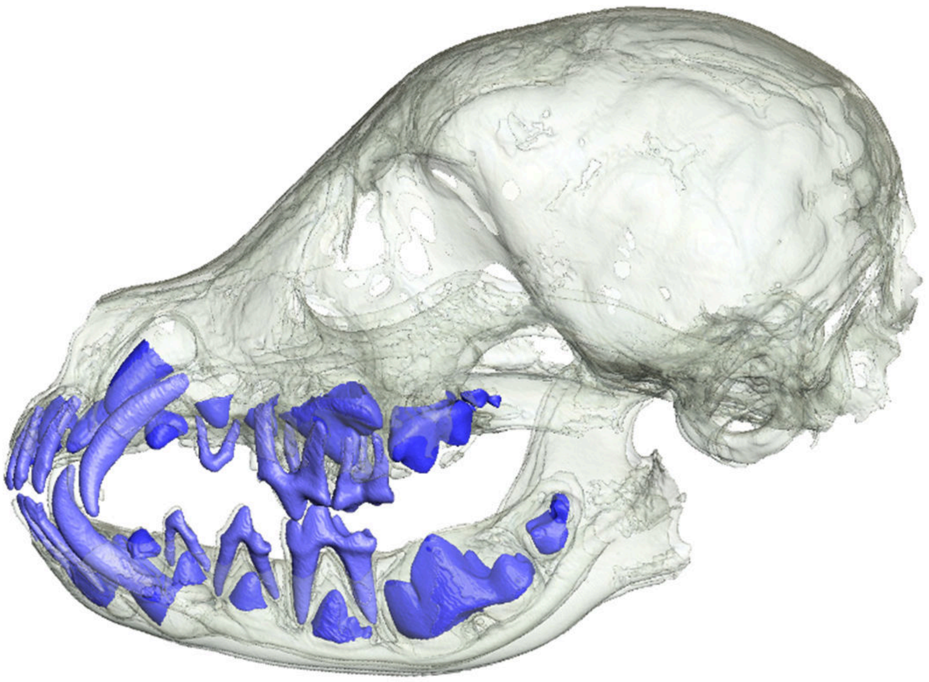

Lateral view

Supplementary Figure S8. Skull MAM1500, juvenile, coated, mixed dentition

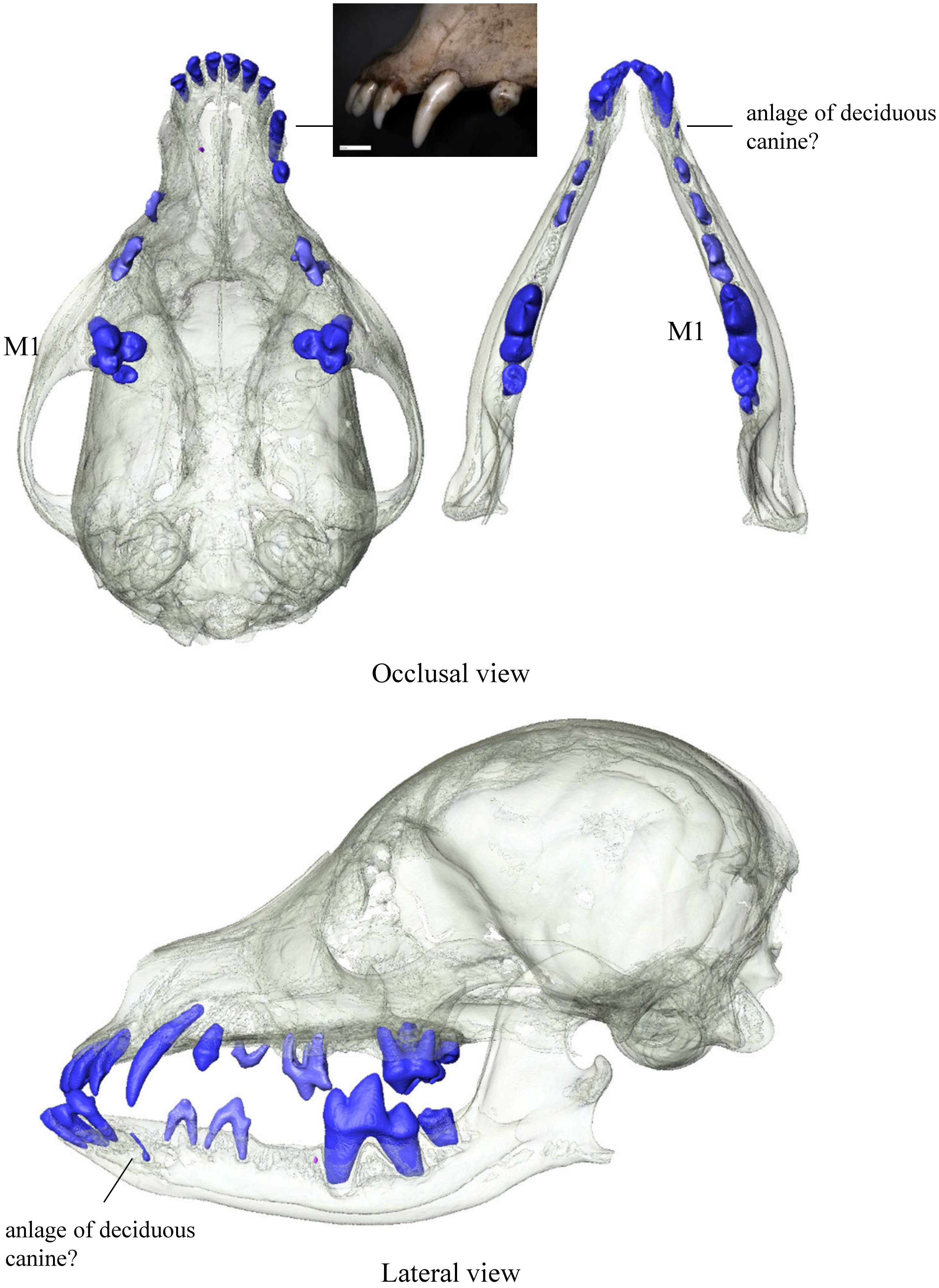

Supplementary Figure S9. Skull MAM1488, adult, hairless, agenesis of permanent premolars and canines

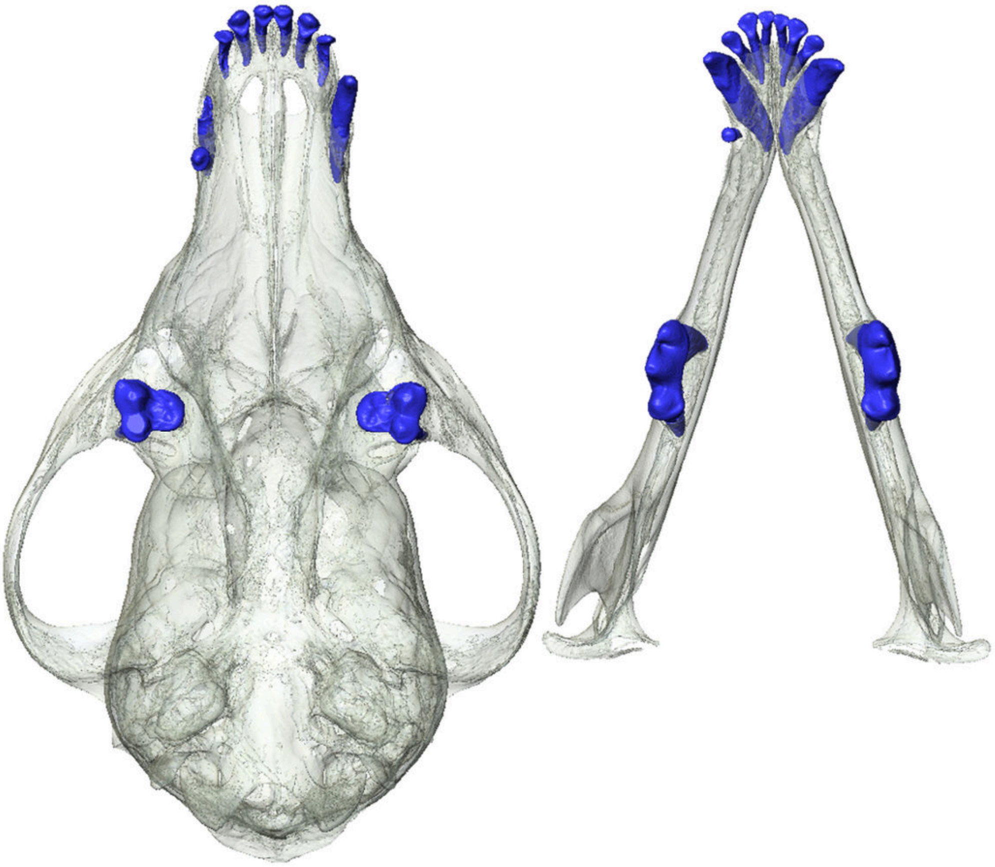

Occlusal view

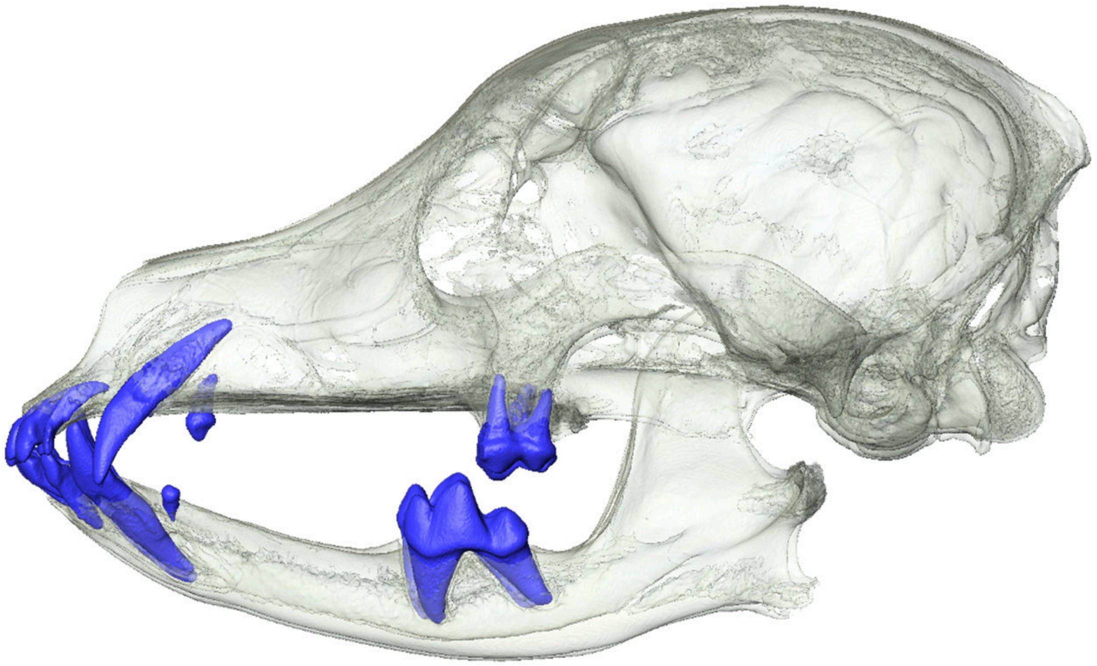

Lateral view

Supplementary Figure S10. Skull MAM1490, adult, hairless, agenesis of permanent premolars and canines

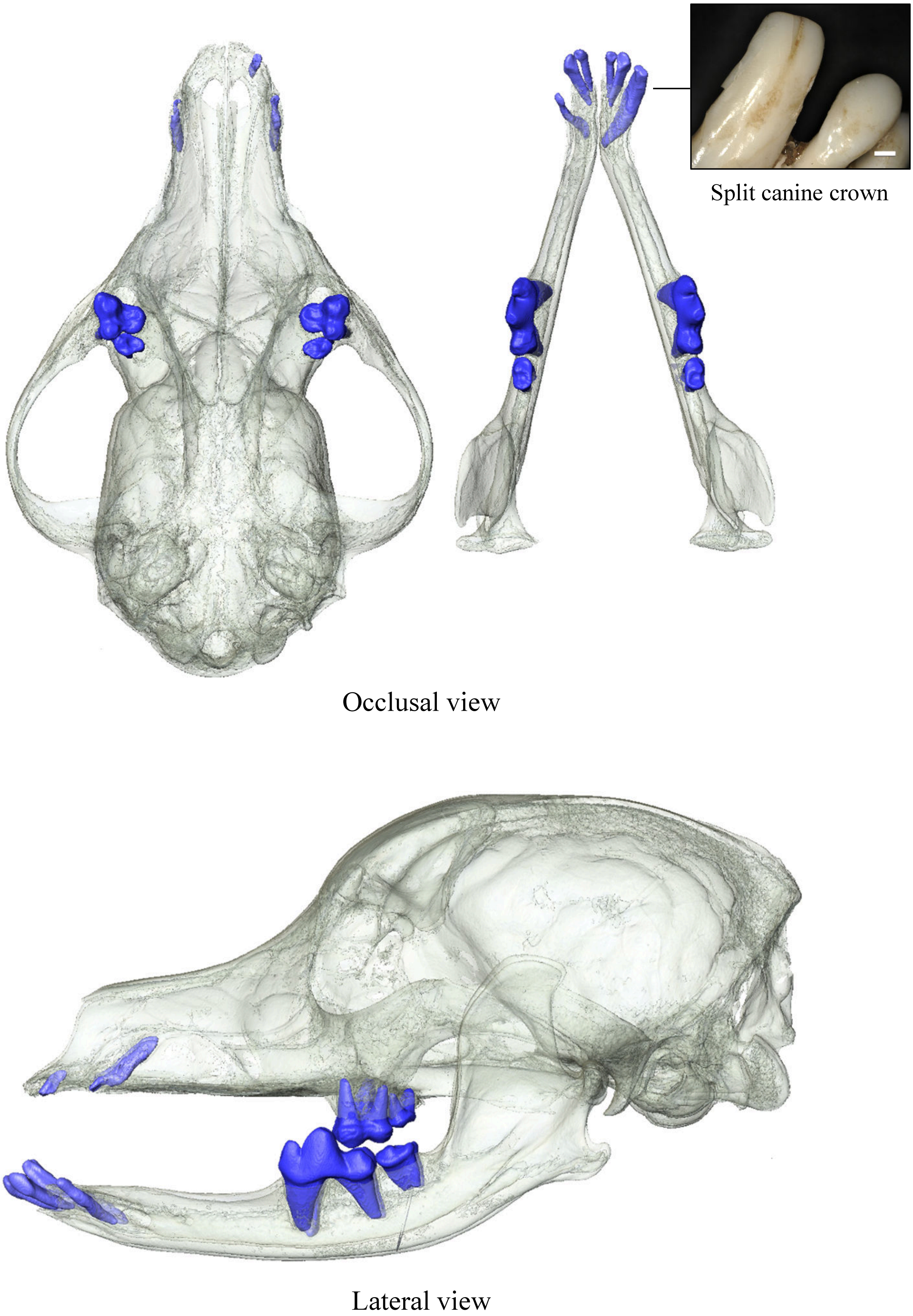

Supplementary Figure S11. Skull MAM1494, adult, hairless, agenesis of permanent incisors, premolars and canines

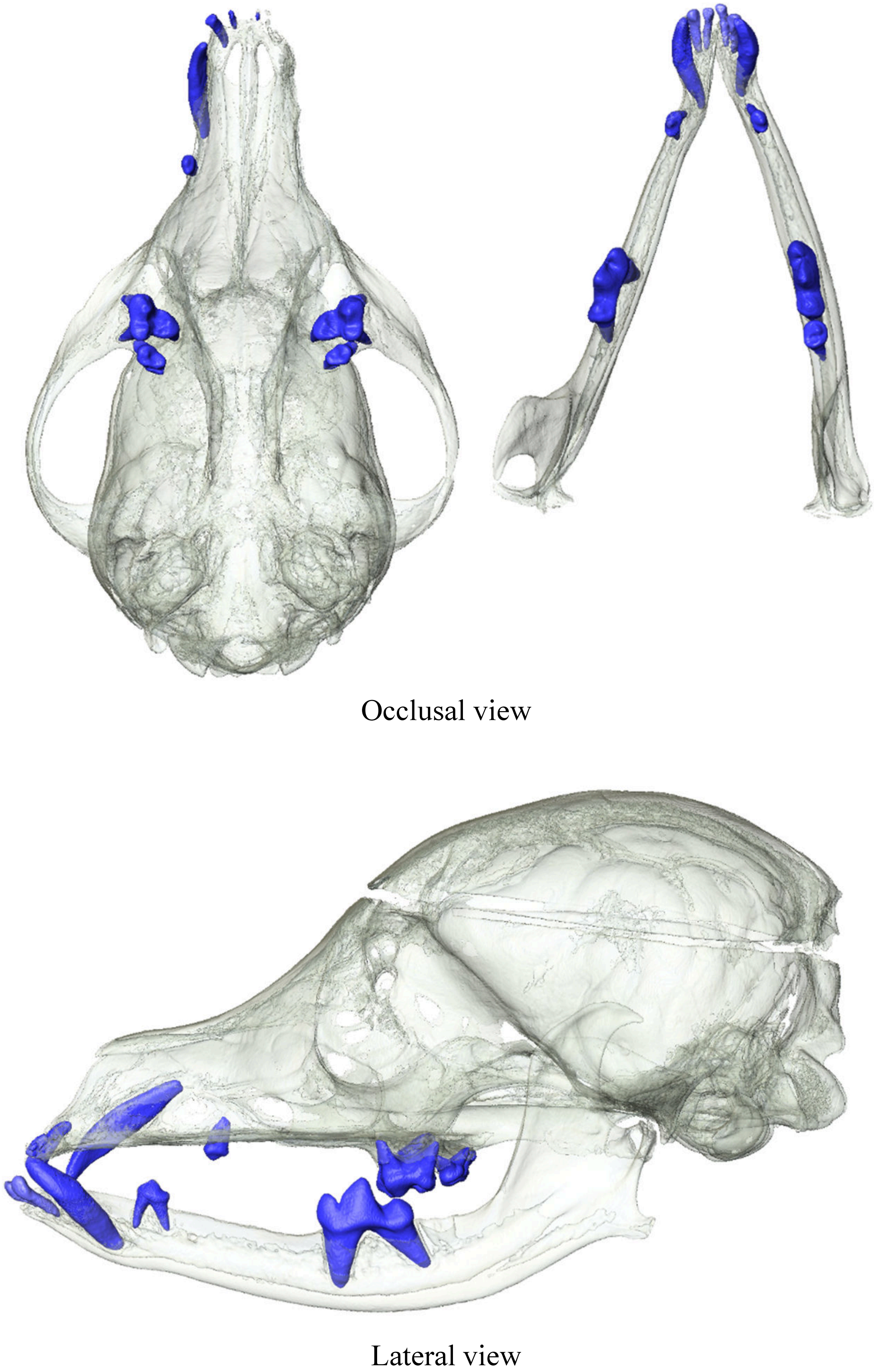

Supplementary Figure S12. Skull MAM1496, adult, hairless, agenesis of permanent incisors, premolars and canines

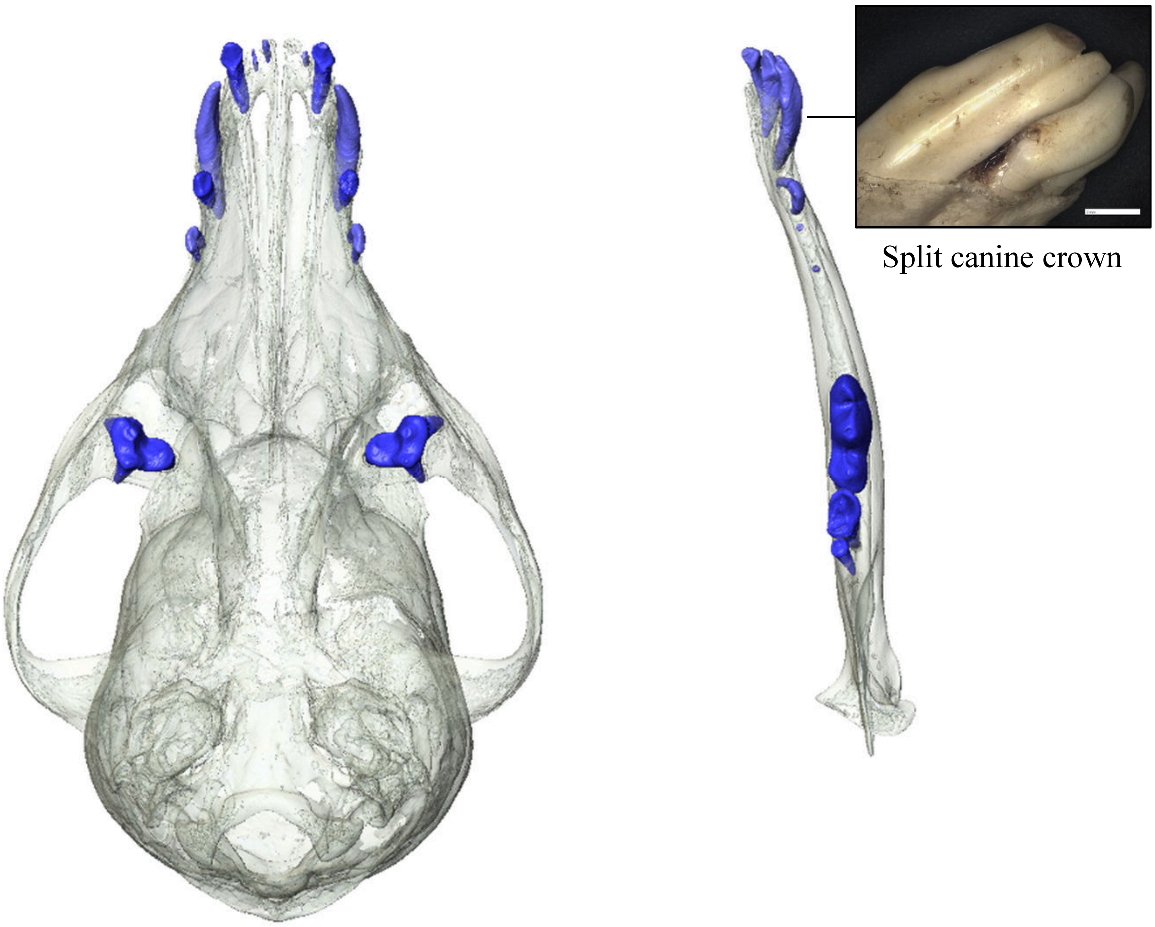

Occlusal view

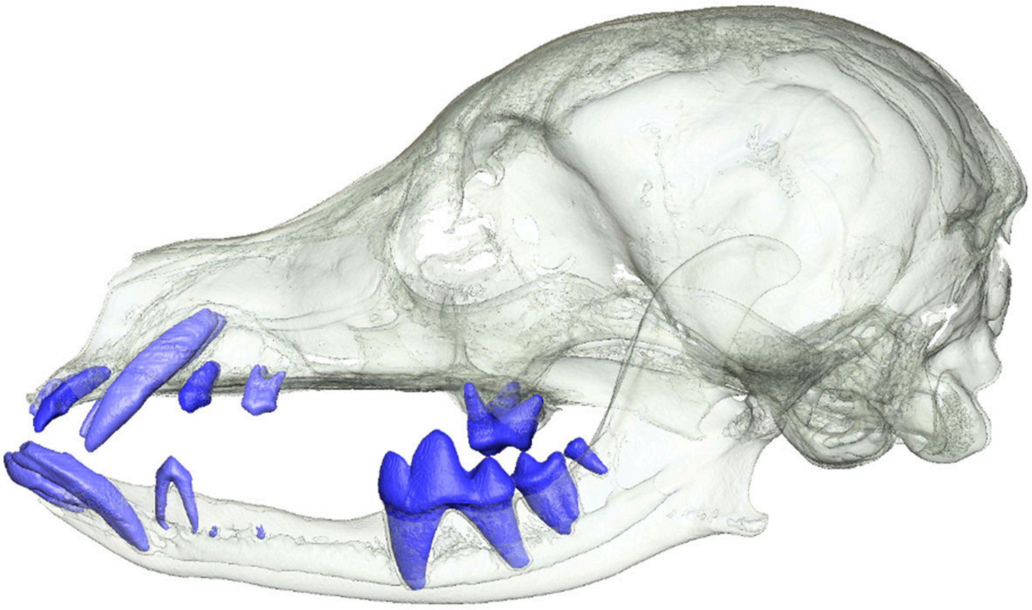

Lateral view

Supplementary Figure S13. Skull MAM1497, adult, hairless, agenesis of permanent incisors, premolars and canines.

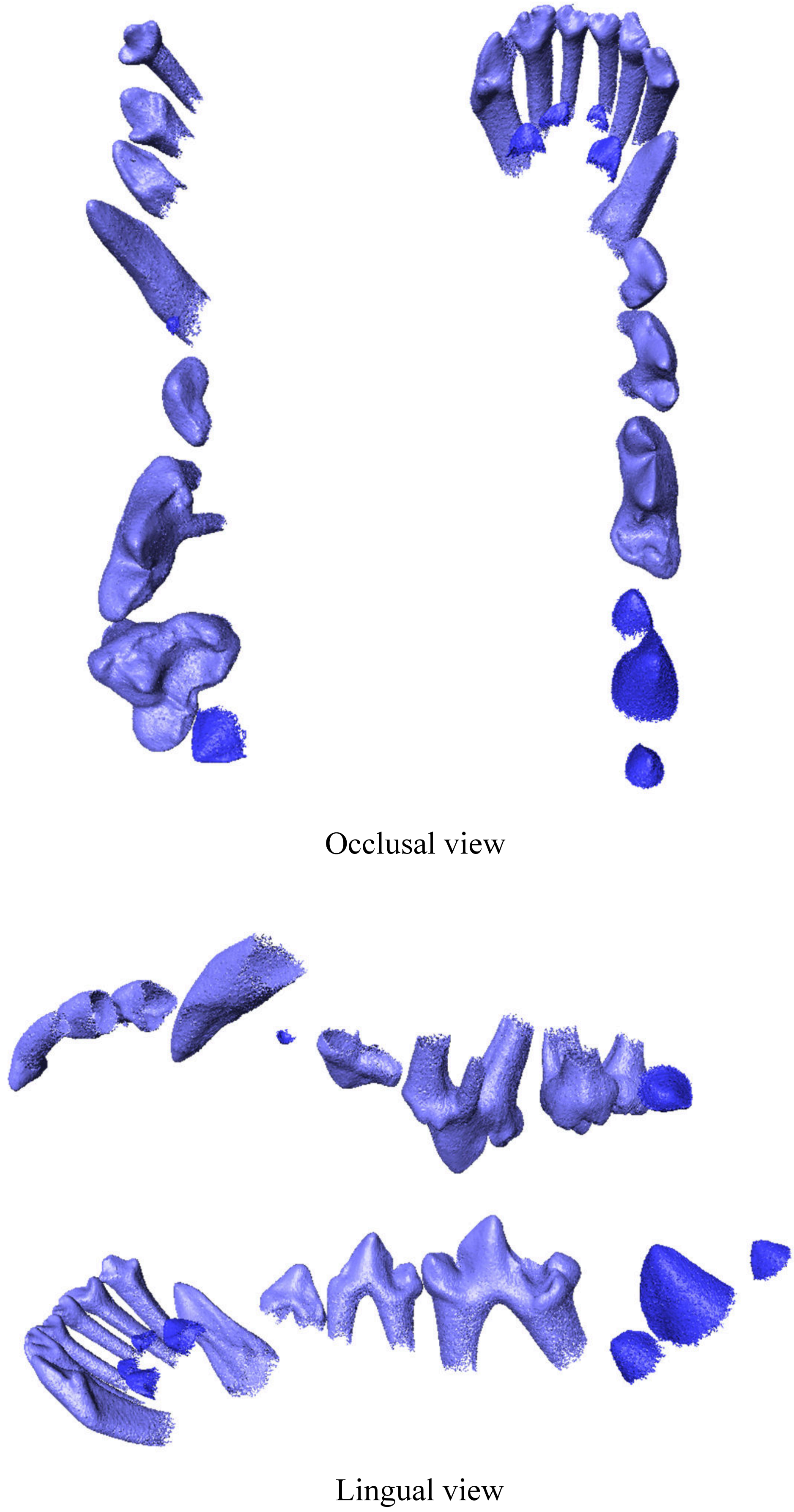

Supplementary Figure S14. Skull MAM2419, juvenile, hairless, mixed dentition

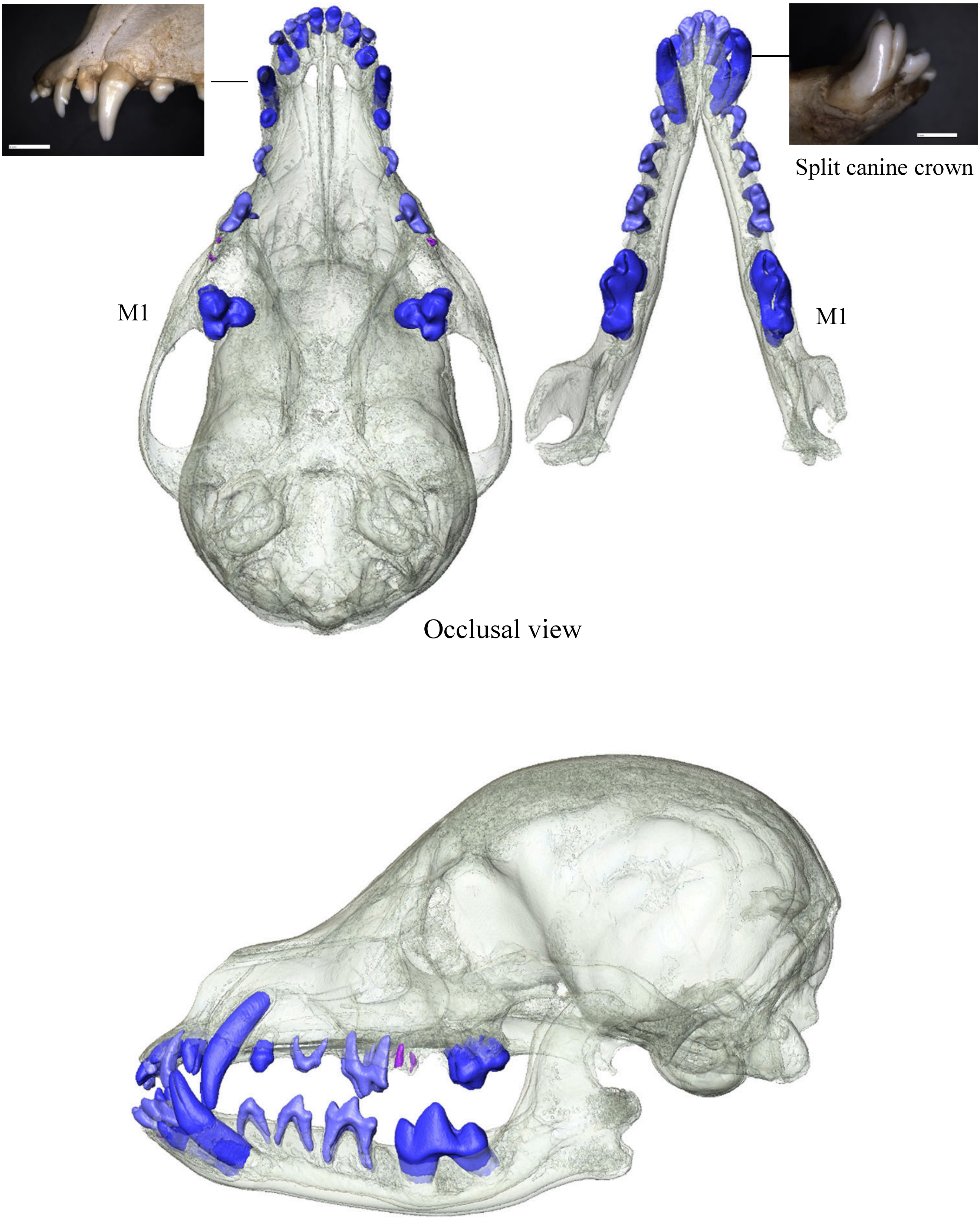

Supplementary Figure S15. Skull MAM1489, juvenile, patchy hair coat, permanent dentition erupted and roots in development

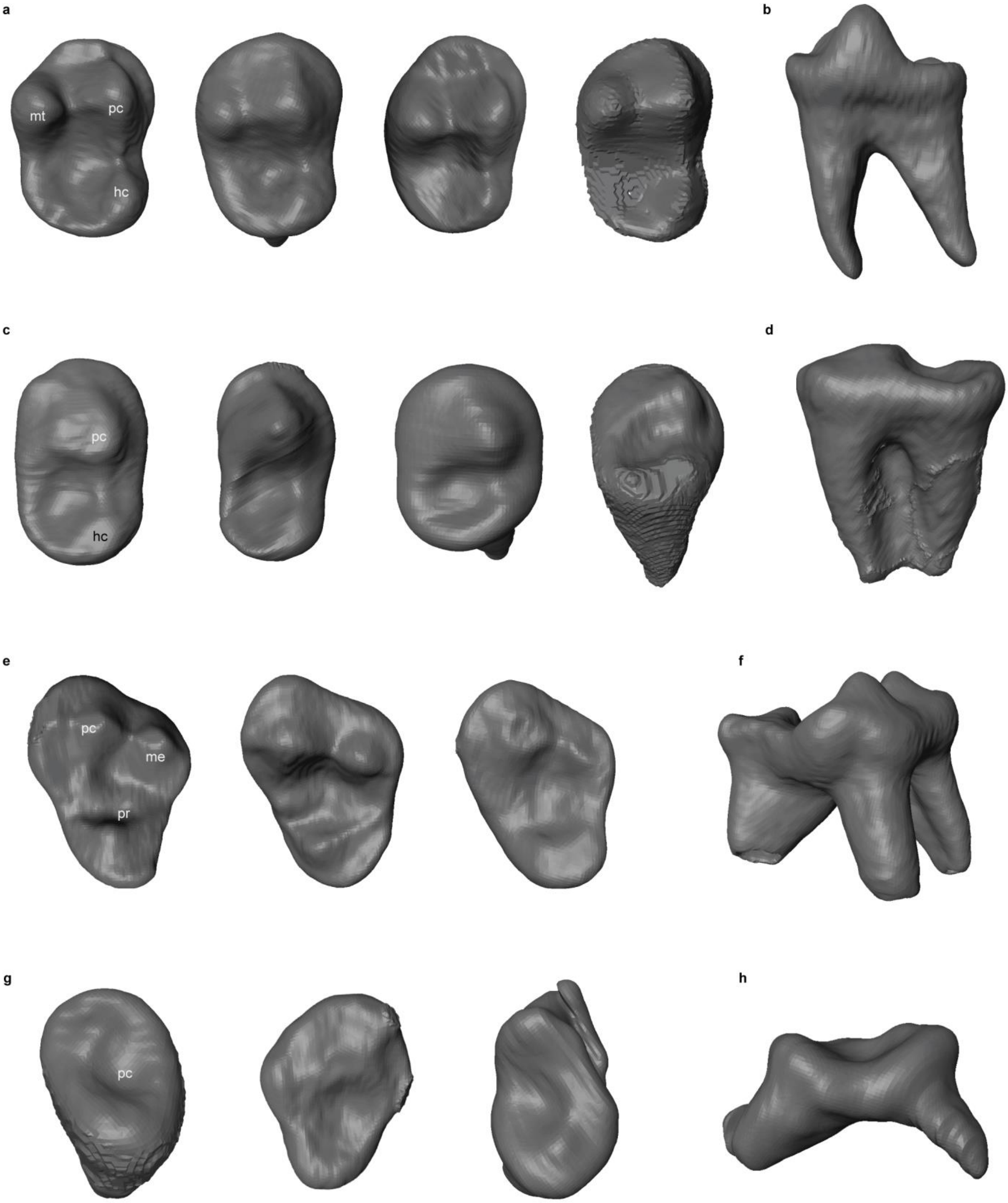

Supplementary Fig. S16: Cusp and root morphology in lower and upper second molars (M_2 and M^2, respectively) in coated and hairless dog sample. a, Occlusal view of M_2 in *FOXI3* +/+ dogs (hc=hypoconid, mt=metaconid, pc=protoconid). b, lateral view of double-rooted M_2 in *FOXI3* +/+ dog. c, Occlusal view of M_2 in *FOXI3* +/- dog with loss of metaconid and reduced hypoconid. d, Lateral view of M_2 with fused roots in *FOXI3* +/- dog. e, Occlusal view of M^2 in *FOXI3* +/+ dogs (me=metacone, pc=paracone, pr=protocone). f, lateral view of three-rooted M^2 in *FOXI3* +/+ dog. g, Occlusal view of M^2 in *FOXI3* +/- dogs with loss of metacone and protoccone. h, lateral view of two-rooted M^2 in *FOXI3* +/- dog.

Supplementary Table S1. Mandibular and maxillary first molar metrics in mm

Accession number	Coat	M_1 md* length	M_1 bl* width	side	M^1 md length	M^1 bl width	side
1493	Coated	15.38	5.52	left	-	-	-
1495	Coated	14.52	4.87	left	-	-	-
1500	Coated	16.55	6.19	right	10.29	12.66	right
1491	Coated	15.74	5.76	left	9.91	12.25	left
1492	Coated	16.99	6.14	left	10.22	12.27	left
1499	Coated	15.13	5.55	right	8.8	9.24	right
1501	Coated	18.11	6.4	left	-	-	-
1488	Hairless	13.75	5.15	right	8.41	9.6	right
1490	Hairless	16.27	6.23	left	10.28	12.17	left
1494	Hairless	16.71	6.02	left	9.94	11.58	left
1496	Hairless	13.1	4.82	left	7.61	9.48	left
1497	Hairless	15.46	5.24	right	6.92	9.6	right
1489	Patchy	15.19	6.1	left	9.29	9.57	left

*md= mesiodistal, bl=buccolingual

Table S2. Relative talonid height of M1

	Trigonid height (mm)	Talonid height (mm)	Rel. talonid height
Coated dogs			
MAM1493	7.72	3.82	0.49
MAM1495	6.23	2.16	0.35
MAM1500	9.65	5.16	0.53
MAM1491	8.51	4.13	0.49
MAM1492	9.01	4.97	0.55
MAM1499	6.67	3.14	0.47
MAM1501	9.38	4.26	0.45
Hairless dogs			
MAM2419	5.98	2.13	0.36
MAM1488	7.81	3.64	0.47
MAM1490	9.19	5.19	0.56
MAM1494	8.07	3.48	0.43
MAM1496	6.39	3.59	0.56
MAM1497	7.92	4.51	0.57
MAM1489*	8.33	4.53	0.54

Relative talonid height is calculated as the ratio of protoconid cusp height (trigonid) and the hypoconid cusp height (talonid). * patchy hair coat
